# The evolutionary history of holometabolous insects inferred from transcriptome-based phylogeny and comprehensive morphological data

**DOI:** 10.1186/1471-2148-14-52

**Published:** 2014-03-20

**Authors:** Ralph S Peters, Karen Meusemann, Malte Petersen, Christoph Mayer, Jeanne Wilbrandt, Tanja Ziesmann, Alexander Donath, Karl M Kjer, Ulrike Aspöck, Horst Aspöck, Andre Aberer, Alexandros Stamatakis, Frank Friedrich, Frank Hünefeld, Oliver Niehuis, Rolf G Beutel, Bernhard Misof

**Affiliations:** 1 Zoologisches Forschungsmuseum Alexander Koenig, Abteilung Arthropoda, Adenauerallee 160, 53113 Bonn, Germany; 2 Zoologisches Forschungsmuseum Alexander Koenig, Zentrum für Molekulare Biodiversitätsforschung (zmb), Adenauerallee 160, 53113 Bonn, Germany; 3 CSIRO Ecosystem Sciences, Australian National Insect Collection, Clunies Ross Street, Acton ACT 2601, Australia; 4 Rutgers University, Department of Ecology, Evolution and Natural Resources, New Brunswick, NJ 08901, USA; 5 Naturhistorisches Museum Wien, 2. Zool. Abteilung, Burgring 7, 1010 Vienna, Austria; 6 Department of Evolutionary Biology, University of Vienna, Althanstraße 14, 1090 Vienna, Austria; 7 Institut für Spezifische Prophylaxe und Tropenmedizin, Medizinische Parasitologie, Medizinische Universität Wien (MUW), Kinderspitalgasse 15, 1090 Vienna, Austria; 8 Heidelberg Institute for Theoretical Studies (HITS), Scientific Computing Group, Schloss-Wolfsbrunnenweg 35, 69118 Heidelberg, Germany; 9 Karlsruher Institut für Technologie, Fakultät für Informatik, Postfach 698076128 Karlsruhe, Germany; 10 Biozentrum Grindel und Zoologisches Museum Hamburg, Universität Hamburg, Martin-Luther-King-Platz 3, 20146 Hamburg, Germany; 11 Entomology Group, Institut für Spezielle Zoologie und Evolutionsbiologie mit Phyletischem Museum, Friedrich-Schiller-Universität Jena, Erbertstraße. 1, 07743 Jena, Germany

## Abstract

**Background:**

Despite considerable progress in systematics, a comprehensive scenario of the evolution of phenotypic characters in the mega-diverse Holometabola based on a solid phylogenetic hypothesis was still missing. We addressed this issue by *de novo* sequencing transcriptome libraries of representatives of all orders of holometabolan insects (13 species in total) and by using a previously published extensive morphological dataset. We tested competing phylogenetic hypotheses by analyzing various specifically designed sets of amino acid sequence data, using maximum likelihood (ML) based tree inference and Four-cluster Likelihood Mapping (FcLM). By maximum parsimony-based mapping of the morphological data on the phylogenetic relationships we traced evolutionary transformations at the phenotypic level and reconstructed the groundplan of Holometabola and of selected subgroups.

**Results:**

In our analysis of the amino acid sequence data of 1,343 single-copy orthologous genes, Hymenoptera are placed as sister group to all remaining holometabolan orders, *i.e.*, to a clade Aparaglossata, comprising two monophyletic subunits Mecopterida (Amphiesmenoptera + Antliophora) and Neuropteroidea (Neuropterida + Coleopterida). The monophyly of Coleopterida (Coleoptera and Strepsiptera) remains ambiguous in the analyses of the transcriptome data, but appears likely based on the morphological data. Highly supported relationships within Neuropterida and Antliophora are Raphidioptera + (Neuroptera + monophyletic Megaloptera), and Diptera + (Siphonaptera + Mecoptera). ML tree inference and FcLM yielded largely congruent results. However, FcLM, which was applied here for the first time to large phylogenomic supermatrices, displayed additional signal in the datasets that was not identified in the ML trees.

**Conclusions:**

Our phylogenetic results imply that an orthognathous larva belongs to the groundplan of Holometabola, with compound eyes and well-developed thoracic legs, externally feeding on plants or fungi. Ancestral larvae of Aparaglossata were prognathous, equipped with single larval eyes (stemmata), and possibly agile and predacious. Ancestral holometabolan adults likely resembled in their morphology the groundplan of adult neopteran insects. Within Aparaglossata, the adult’s flight apparatus and ovipositor underwent strong modifications. We show that the combination of well-resolved phylogenies obtained by phylogenomic analyses and well-documented extensive morphological datasets is an appropriate basis for reconstructing complex morphological transformations and for the inference of evolutionary histories.

## Background

Holometabola (or Endopterygota) are, given their evolutionary age, by far the most species-rich subgroup of insects (Hexapoda) and comprise more than 60% of all described metazoan species [1]. Within the Holometabola, the mega-diverse orders Coleoptera (beetles), Diptera (midges, mosquitos, and flies), Lepidoptera (moths and butterflies), and Hymenoptera (sawflies, bees, wasps, and ants) comprise together almost 800,000 species [2] and therefore more than 95% of the total species diversity of the entire lineage. The smaller orders are Neuroptera (lacewings), Megaloptera (alderflies and dobsonflies), Raphidioptera (snakeflies), Trichoptera (caddisflies), Mecoptera (scorpionflies and relatives), and Siphonaptera (fleas). Complete metamorphosis, which is characterized by the presence of a more or less inactive and non-feeding pupal stage between a feeding larva and a reproducing adult, is the most striking difference between Holometabola and other hexapods. Whereas the monophyly of Holometabola and of all its orders (with few exceptions, see below) has been consistently recovered (*e.g.*, [1,3]), the interordinal relationships are still insufficiently resolved. This impedes our understanding of the ancestral holometabolan morphology and life history and the modifications that occurred during the subsequent diversification of this highly successful lineage.

A reliable reconstruction of evolutionary transformations within Holometabola requires a well-founded hypothesis of the phylogenetic relationships of the major included groups. The first comprehensive reconstruction of holometabolan phylogenetic relationships was presented by Hennig [4], although a substantial contribution had already been made earlier by Hinton [5]. Alternative concepts to Hennig’s proposal were presented by Rasnitsyn and Quicke [6] and Kukalová-Peck and Lawrence [7], with the main difference that Hymenoptera were not placed as sister group of Mecopterida (Diptera, Siphonaptera, and Mecoptera (= Antliophora), and Lepidoptera and Trichoptera (= Amphiesmenoptera)) (as in [4] and, *e.g.*, [1,8,9]), but as the first diverging extant holometabolan insect order. A distinctly different view was presented by Wheeler and colleagues [10] (see also [11,12]): they discussed a sister group relationship between Hymenoptera and Mecopterida (as in Hennig’s concept), a sister group relationship between Strepsiptera and Diptera (Halteria), and paraphyletic Mecoptera, with the mecopteran Boreidae as sister group of Siphonaptera. Based on entirely new molecular and morphological datasets, Wiegmann et al. [13], McKenna and Farrell [14], and Beutel et al. [15] (see also [16]) congruently revived the view that Hymenoptera are sistergroup of all remaining Holometabola; Strepsiptera were recovered as closely related to Coleoptera, and Mecoptera were found monophyletic. Recently, these hypotheses gained additional support by a phylogenetic analysis of nucleotide sequence data from whole genome sequencing projects [17]. However, several interordinal relationships within Holometabola remained elusive. Despite remarkable progress, the genomic depth of published molecular sequence data, which potentially offers a plethora of phylogenetically informative characters, is still very low: large-scale transcriptome or genome data have been only available for representatives of less than half of all recognized holometabolan orders, with most studies so far dealing with model species. Consequently, the aim of our study was to present the first reconstruction of holometabolan relationships based on transcriptomic data of representatives of all currently recognized orders.

In this study, we address the following phylogenetic questions:

1. Are Hymenoptera the sister group of Mecopterida (Antliophora and Amphiesmenoptera) or of all other holometabolan insect lineages (*e.g.*, [4]*versus*[13])?

2. Are Neuropteroidea (Neuropterida, Coleoptera, and Strepsiptera) monophyletic? Neuropteroidea were found monophyletic by Wiegmann et al. [13] but not found by Wheeler et al. [10], Kukalová-Peck and Lawrence [7], and Beutel et al. [15].

3. Are Megaloptera monophyletic? and 4. Are Neuroptera and Megaloptera sister groups? Proposed relationships of the groups of Neuropterida (Megaloptera, Neuroptera, and Raphidioptera) are incongruent, and nearly all possible topological arrangements concerning this problem have been published over the last years (see, *e.g.*, [1,3,15,18-21]).

5. Are Coleopterida (Coleoptera and Strepsiptera) monophyletic? The whole genome-based analyses by Niehuis et al. [17] inferred Strepsiptera as sister group of Coleoptera, but did not include representatives of Neuropterida.

6. Are Mecopterida monophyletic? This group was neither found monophyletic by Kukalová-Peck and Lawrence [7] nor by some of the analyses in Beutel et al. [15], but was monophyletic in Wiegmann et al. [13], though not well supported.

7. What are the phylogenetic relationships within Antliophora? Contradicting phylogenetic relationships among Diptera, Mecoptera, and Siphonaptera have been published, and the monophyly of Mecoptera has been questioned (see above, and [8,10,13,15]).

In order to address the above questions, we generated transcriptomic data of at least one representative of each holometabolan order. For transcriptome sequencing, we selected species mostly characterized by plesiomorphic morphological character conditions and representing taxa that presumably diverged early in the evolutionary history of each group (see [15]). In our molecular phylogenetic analyses, we used specific decisive datasets for each of our phylogenetic questions. Following the arguments put forth by Dell’Ampio et al. [22], a dataset is deemed “decisive” if information of each gene is available from each taxonomic group of interest and thus can contribute to resolving the relationships among these groups. In addition to maximum likelihood (ML) based tree inference, we applied Four-cluster Likelihood Mapping (FcLM) [23] to study potential incongruent signal in our datasets that might not be revealed by a phylogenetic multi-species tree.

We mapped a comprehensive set of morphological data [15] on the transcriptome-based phylogeny, and addressed the following issues regarding the evolutionary history of Holometabola:

•Major morphological features of the ancestral larva and the ancestral adult of Holometabola (groundplan) (*e.g*., larval eyes, legs, prognathous *versus* orthognathous head; adult prognathous *versus* orthognathous head, size of pterothoracic segments, eyes)

•Ancestral larval and adult life habits of Holometabola (*e.g*., diet, phytophagy/fungivory *versus* carnivory)

•Major transformations of larval and adult characters within Holometabola (*e.g.,* flight apparatus transformations: shift of segment and wing size, wing coupling mechanisms; modifications of oviposition strategy)

•Ancestral mode of ontogenetic development of Holometabola (*e.g*., pupal characters)

In summary, we aimed to trace evolutionary changes of phenotypic features and to reconstruct groundplans for Holometabola and well-established clades within the Holometabola tree. An evolutionary history based on a solid phylogenetic background represents an important step toward a better understanding of the unparalleled diversification of this exceptional group of organisms.

## Results and discussion

### The phylogeny of Holometabola

**Figure 1:**
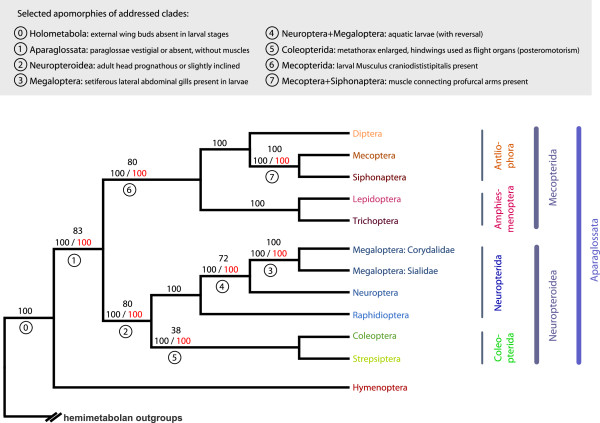
**Combined and simplified cladogramm of holometabolan insect relationships, with selected autapomorphies for the clades addressed in this study.** The topology is taken from the ML tree inferred from dataset 1 (*i.e.*, the complete datamatrix). (1) Bootstrap support (BS) (bottom, black) is derived from 72 bootstrap replicates (MRE-based bootstopping criterion) of dataset 1. (2) BS values for the specific phylogenetic relationship (bottom, red) are derived from ML tree inferences from the seven specific decisive datasets 1 to 7. (3) relative support [%] values for the specific phylogenetic relationship (top) are derived from the Four-cluster Likelihood Mapping (FcLM) with the seven specific decisive datasets. Apomorphies are selected from the full lists of reconstructed groundplan characters (see Additional file 4, Chapter 5).

We analyzed a total of 1,343 1:1 orthologous genes (*i.e.*, groups of orthologous sequences, also called ortholog groups (OGs)) and, by including also published data, data from a total of 88 species (Table 1). The seven specifically designed decisive datasets that we analyzed to address our seven phylogenetic questions each consisted of a subset of taxa and genes from the complete dataset, except for dataset 1 which is identical to the complete dataset. The seven questions, the taxonomic groups that we selected as relevant for answering the questions, and the numbers of species and OGs for each dataset are shown in Table 2. For each dataset we performed 1) ML tree reconstruction, and 2) Four-cluster Likelihood Mapping (FcLM) (see Table 3). Results are summarized in Figure 1 (see Additional file 1: Figures S1-S7 for presence and absence of genes in the datasets, Additional file 2: Figures S8-S15 for the full phylogenetic trees, and Additional file 3: Figures S17-S25 for the full results of the FcLM).

**Table 1:** Holometabola species, for which data were newly sequenced

**Order**	**Family**	**Species**	**No. of contigs**	**No. of OGs**
Hymenoptera	Xyelidae	*Xyela alpigena* (Strobl, 1895)	9,931	471
Raphidioptera	Raphidiidae	*Raphidia ariadne* Aspöck & Aspöck, 1964	29,636	983
Neuroptera	Nevrorthidae	*Nevrorthus apatelios* Aspöck, Aspöck & Hölzel, 1977	17,673	695
Megaloptera	Sialidae	*Sialis lutaria* (Linnaeus, 1758)	14,200	801
Megaloptera	Corydalidae	Corydalinae sp.	60,455	1,109
Coleoptera	Cupedidae	*Priacma serrata* (Leconte, 1861)	18,808	868
Coleoptera	Carabidae	*Carabus granulatus* (Linnaeus, 1758)	55,582	1,159
Strepsiptera	Mengenillidae	*Mengenilla moldrzyki* Pohl et al., 2012	60,642	999
Lepidoptera	Micropterigidae	*Micropterix calthella* (Linné, 1761)	137,093	969
Trichoptera	Philopotamidae	*Philopotamus ludificatus* McLachlan, 1878	24,628	914
Diptera	Tipulidae	*Tipula maxima* Poda, 1761	24,724	938
Siphonaptera	Pulicidae	*Archaeopsylla erinacei* (Bouché, 1835)	35,270	1,191
Mecoptera	Nannochoristidae	*Nannochorista philpotti* (Tillyard, 1917)	44,935	1,212

Shown are taxonomic classification, number of contigs after assembly (only contigs longer than 200 bp after removal of suspicious sequences are considered, according to the NCBI guidelines (VecScreen)), and number of assigned single-copy orthologous genes in the complete dataset (after redundancy and outlier check, see Methods section).

**Table 2:** The seven datasets, designed to address seven phylogenetic questions

**Dataset**	**Addressed phylogenetic question**	**Covered subgroups/FcLM clusters (4 clusters per analysis)**	**No. of species**	**No. of OGs**	**Alignment length (aa)**	**Coverage [%] all species**	**Coverage [%] addressed groups**
Dataset 1 (complete dataset)	Position of Hymenoptera?	1) Hymenoptera	88	1,343	662,107	61.1	100
		2) outgroup taxa					
		3) Mecopterida					
		4) Neuropteroidea					
Dataset 2	Are Neuropteroidea monophyletic?	1) Neuropterida	71	1,303	643,051	65.0	100
		2) Mecopterida					
		3) Coleopterida					
		4) Hymenoptera					
Dataset 3	Are Megaloptera monophyletic?	1) Raphidioptera	4	358	174,065	100	100
		2) Corydalidae					
		3) Sialidae					
		4) Neuroptera					
Dataset 4	Are Neuroptera and Megaloptera sister groups?	1) Raphidioptera	71	540	242,820	72.9	100
		2) Megaloptera					
		3) Neuroptera					
		4) remaining holometabolans					
Dataset 5	Are Coleopterida monophyletic?	1) Neuropterida	71	972	505,528	66.2	100
		2) Strepsiptera					
		3) Coleoptera					
		4) remaining holometabolans					
Dataset 6a	a) Are Mecopterida monophyletic? or	a) 1) Antliophora	71	1,343	662,107	64.3	100
Dataset 6b	b) Are Antliophora + Coleopterida monophyletic?	2) Amphiesmenoptera					
		3) Neuropteroidea					
		4) remaining holometabolans					
		b) 1) Antliophora					
		2) Amphiesmenoptera					
		3) Coleopterida					
		4) remaining holometabolans					
Dataset 7	Relationships within Antliophora?	1) Diptera	71	1,101	557,276	66.5	100
		2) Siphonaptera					
		3) Mecoptera					
		4) remaining holometabolans					

For each dataset, we selected four taxonomic groups (clusters), assigned species to one of the groups, and extracted only those ortholog groups (OGs) that contained a sequence of at least one representative of each group. All species that were not assigned to either of the groups were excluded. Coverage [%] all species: Coverage of the dataset in terms of presence of OGs considering all species. Coverage [%] addressed groups: Coverage of the dataset in terms of presence of OGs considering the four groups defined for each dataset, which is, by definition, 100%.

**Table 3:** FcLM Results

**Dataset**	**Possible unambiguous topologies**	**No. of drawn quartets**	**Support T1 [%] 1,2 | 3,4**	**Support T2 [%] 1,3 | 2,4**	**Support T3 [%] 1,4 | 2,3**
Dataset 1 (complete dataset)	**T1: Hymenoptera,outgroup taxa | Mecopterida, Neuropteroidea**	142,800	**83**	8	8
	T2: Hymenoptera, Mecopterida | outgroup taxa, Neuropteroidea				
	T3: Hymenoptera, Neuropteroidea | outgroup taxa, Mecopterida				
Dataset 2	T1: Neuropterida, Mecopterida | Coleopterida, Hymenoptera	20,160	8	**80**	11
	**T2: Neuropterida, Coleopterida | Mecopterida, Hymenoptera**				
	T3: Neuropterida, Hymenoptera | Mecopterida, Coleopterida				
Dataset 3	T1: Raphidioptera, Corydalidae | Sialidae, Neuroptera	1	0	0	**100**
	T2: Raphidioptera, Sialidae | Corydalidae, Neuroptera				
	**T3: Raphidioptera, Neuroptera | Corydalidae, Sialidae**				
Dataset 4	T1: Raphidioptera, Megaloptera | Neuroptera, remaining holometabolans	134	25	1	**72**
	T2: Raphidioptera, Neuroptera | Megaloptera, remaining holometabolans				
	**T3: Raphidioptera, remaining holometabolans | Megaloptera, Neuroptera**				
Dataset 5	T1: Neuropterida,Strepsiptera | Coleoptera,remaining holometabolans	1,220	6 (8)	**55 (53)**	38 (38)
	**T2: Neuropterida, Coleoptera | Strepsiptera,remaining holometabolans**				
	T3: Neuropterida,remaining holometabolans | Strepsiptera, Coleoptera				
Dataset 6a	**T1: Antliophora, Amphiesmenoptera | Coleopterida, remaining holometabolans**	80,640	**80**	14	5
	T2: Antliophora, Coleopterida | Amphiesmenoptera, remaining holometabolans				
	T3: Antliophora, remaining holometabolans | Amphiesmenoptera, Coleopterida				
Dataset 6b	**T1: Antliophora, Amphiesmenoptera | Coleopterida, remaining holometabolans**	57,600	**79**	15	5
	T2: Antliophora, Coleopterida | Amphiesmenoptera, remaining holometabolans				
	T3: Antliophora, remaining holometabolans | Amphiesmenoptera, Coleopterida				
Dataset 7	T1: Diptera, Siphonaptera | Mecoptera, remaining holometabolans	1,034	0	0	**100**
	T2: Diptera, Mecoptera | Siphonaptera, remaining holometabolans				
	**T3: Diptera, remaining holometabolans | Siphonaptera, Mecoptera**				

For the four groups (clusters) that were selected for each of the seven datasets, three unambiguous topologies are possible (see Additional file 4, Chapter 3, and Additional file 3: Figure S16). For details which species are included in the groups for each dataset see Additional file 12. The number of drawn quartets is the product of the numbers of species in each group. In bold print: Topology that gained the highest support (support [%]: relative amount of quartets which show predominant support for either T_1_, T_2_ or T_3_). Results of partitioned analyses of dataset 5 in parentheses.

The analysis of dataset 1 yielded Hymenoptera as sister group to all remaining holometabolan orders in both ML tree reconstruction and FcLM (Table 3, Figure 1). This relationship had already been recovered in several multiple gene studies (*e.g.*, [13,14]), and based on whole genome data but a limited taxon sampling [17]. Previously published analyses of morphological data yielded contradictory results, such as for instance Hymenoptera + Mecopterida in Beutel and Gorb [9]*versus* Hymenoptera + remaining holometabolan orders in Beutel et al. [15]. Potential problems of topological artifacts in these analyses that are caused by convergent reductions in many morphological character systems were discussed in detail by Friedrich and Beutel [24] and Beutel et al. [15]. The placement of Hymenoptera as sister group to all remaining holometabolan orders implies that presumptive synapomorphies of Hymenoptera and Mecopterida (*e.g*., single claw of larvae, sclerotized sitophore plate of adults; see [1]) are in fact homoplasies.

Our analyses of dataset 2 yielded monophyletic Neuropteroidea (*i.e.*, a clade comprising Neuropterida, Coleoptera, and Strepsiptera) with maximal support in the ML tree reconstruction and strong support in the FcLM (Table 3, Figure 1). Neuropteroidea was not supported as a clade in Beutel et al. [15], but was found monophyletic in many previous studies [1,8,9,13,14,25], even though in most cases with weak or without support.

We did not find any signal for paraphyletic Megaloptera as discussed by Beutel et al. [15] and Winterton et al. [26] (dataset 3, Table 3, Figure 1). Within Neuropterida, our ML analyses maximally supported a sister group relationship between Raphidioptera and Neuroptera + Megaloptera, which was also supported by more than 2/3 of all quartets in the FcLM (dataset 4, Table 3, Figure 1). Phylogenetic relationships among neuropterid orders have been discussed controversially with two alternative hypotheses: Raphidioptera + Megaloptera being monophyletic (*e.g.*, [8,9,13-15,27]) or Neuroptera + Megaloptera being monophyletic (*e.g.*, [18,19,25,28,29]). Our results strongly support the latter hypothesis.

Analysis of dataset 5 yielded ambiguous results with respect to a possible clade comprising Coleoptera and Strepsiptera (Coleopterida) (Table 3, Figure 1). Resolving this longstanding problem is difficult due to the extremely modified morphology (*e.g.*, [30]) and the distinctly derived genomic features [17,31] of the endoparasitic Strepsiptera (“the Strepsiptera problem“, [1]; “insects from outer space”, [32]). In most recent contributions, evidence was found for monophyletic Coleopterida (*e.g.*, [13-15,17]). However, the studies based on molecular data remained ambiguous in their results. Coleopterida were not supported by all datasets analyzed by McKenna and Farrell [14]. The results of Wiegmann et al. [13] were based on a relatively small set of genes and showed only weak support for this clade. Niehuis et al. [17] analyzed whole genome nucleotide sequences of holometabolous insects and found well-supported Coleopterida but the taxon sampling did not include any neuropterid orders. In our study, Coleopterida is supported in the ML tree (with maximal bootstrap support), but not in the FcLM analyses (Table 3, Figure 1). In the ML tree, Strepsiptera are placed within Coleoptera (like in some of the trees of McKenna and Farrell [14]), however, with poorly supported relationships (Additional file 2: Figure S12). We further analyzed whether the incongruence between ML tree reconstruction and FcLM analyses vanished considering partitioned ML and FcLM analyses using different models on different partitions. Partitioned analyses might reduce potential model misspecifications and might yield congruent topologies. However, the incongruence between ML and FcLM analyses did not disappear (Table 3, Additional files 2 and 3). This implies that model misspecifications due to unpartitioned analyses are not the source of incongruence (see also [22] and discussion therein). Apparently, the data and analytical procedures of our study did not yield an unambiguous solution of the question whether or not Coleopterida is a monophyletic group. However, evidence from morphology clearly suggests monophyletic Coleopterida (see also [17]) as the most plausible result.

In order to test the monophyly of Mecopterida, a clade comprising Amphiesmenoptera (Lepidoptera + Trichoptera) and Antliophora (Diptera + Siphonaptera + Mecoptera), we analyzed two versions of dataset 6 to account for two possible hypotheses (dataset 6a, b; Tables 2 and 3). Both analyses recovered monophyletic Mecopterida with strong support (Table 3, Figure 1). Monophyletic Mecopterida, as proposed by Hinton [5] under the name Panorpoidea (or panorpoid complex), was not well supported in Kjer et al. [25] and Wiegmann et al. [13], and only supported in the Bayesian analyses of morphological characters in Beutel et al. [15]. Niehuis et al. [17] found tentative support for this clade based on whole genome data but the incomplete taxon sampling – genomes of Neuropterida, Trichoptera, Siphonaptera, and Mecoptera have not been sequenced yet – diminished the decisiveness of this dataset concerning the question of monophyletic Mecopterida.

Our analyses clearly corroborated the monophyly of Amphiesmenoptera (Trichoptera + Lepidoptera) (Figure 1). However, we did not test this hypothesis with a specifically designed dataset because it has never been seriously disputed [1].

Within Antliophora, which showed maximal bootstrap support in the ML tree, we found a sister group relationship of Mecoptera and Siphonaptera, also with maximal bootstrap support and with maximal support in the FcLM (dataset 7, Table 2, Figure 1). This result corroborates views put forward by Beutel and Gorb [8], McKenna and Farrell [14], and Wiegmann et al. [13], though the clade Mecoptera + Siphonaptera was not well supported in the latter study. A sister group relationship between Diptera and Siphonaptera as retrieved in Beutel et al. ([15], see discussion therein) is highly unlikely based on our analyses.

With this study, we do not contribute to the question whether Mecoptera are a monophyletic group as only one species, *Nannochorista philpotti,* was part of our taxon sampling. However, morphological data [15] and analyses of nine nuclear genes [14] strongly suggest that Mecoptera indeed form a monophyletic group.

In summary, we inferred a solid phylogenetic backbone of Holometabola, with three maximally supported mega-diverse clades Hymenoptera, Neuropteroidea, and Mecopterida, with approximately 135,000, 370,000, and 300,000 described species, respectively. For the well-defined unit comprising Neuropteroidea and Mecopterida we suggest the name Aparaglossata (Figure 1). The name refers to the loss of the paraglossae, one of the most conspicuous apomorphies of the group (see below and Table 4).

**Table 4:** Selection of groundplan characters and apomorphies of Holometabola and of those holometabolan subgroups whose phylogenetic relationships were addressed in this study and whose monophyly was confirmed

**Taxon**	**Characters**
Holometabola	•* Larval head orthognathous
	•* Larval compound eyes simplified but present
	•* Ocelli absent in larvae
	•* Larval tentorium X-shaped
	•* Retractile larval abdominal prolegs absent
	• Larval cerci absent (possible reversal in Strepsiptera [homology uncertain])
	•* Adult head orthognathous
	• Meso- and metasternum invaginated
	• Meso- and metacoxae closely adjacent medially
	• Appearance of fully developed compound eyes including external apparatus in the pupal stage (reversal in Strepsiptera)
	• External wing buds absent in larval stages (partial reversal in Strepsiptera)
Aparaglossata (Holometabola excluding Hymenoptera)	• Larval head prognathous
	• Well-developed larval stemmata
	• Larval tentorium H-shaped
	• Paraglossae vestigial or absent, without muscles
	• Ventral sclerites of segment VIII (gonocoxae and gonapophyses) indistinct (reversals within Neuropterida)
Neuropteroidea § (Neuropterida and Coleopterida)	• Adult head prognathous or slightly inclined (reversal in Neuroptera)
Megaloptera §	• Sensorium on antepenultimate larval antennomere
	• Larval salivary duct strongly narrowed, without recognizable lumen
	• Setiferous lateral abdominal gills present in larvae
Neuroptera + Megaloptera	• Mesothoracic prealare present (also in Amphiesmenoptera)
	• Muscular connection between metafurcal arm and epimeral apophysis
	• Aquatic larvae (with reversal)
Coleopterida (Coleoptera and Strepsiptera)	• Antenna with 9 flagellomeres or less
	• Pronotum and propleuron partly or completely connected (also in Diptera)
	• Metathorax enlarged, hind wings used as flight organs (posteromotorism)
	• Membranous area between mesoscutellum and mesopostnotum present
Mecopterida (Antliophora and Amphiesmenoptera)	• Larval dorsal tentorial arm strongly reduced or absent
	• Less than 3 larval antennomeres (reversal to 3 in some groups)
	• Larval galea and lacinia extensively or completely fused (also missing as separate structures in Neuroptera and Strepsiptera)
	• Larval Musculus craniodististipitalis present
Siphonaptera + Mecoptera §	• Muscle connecting profurcal arms (Musculus profurca-spinalis) present
	• Acanthae of proventriculus close-set, prominently elongated

Plesiomorphic groundplan characters are marked with an asterisk *. For a full list and for apomorphies found for additional subgroups see Additional file 4, Chapter 5. Characters apply to adults if not mentioned otherwise. For groups marked with § behind taxon name, no selection but rather all obtained apomorphies are listed. Groundplan characters and apomorphies were inferred from the morphological datamatrix of Beutel et al. [15] and the interordinal topology of the ML tree of dataset 1 by formal character mapping in Mesquite [33].

Our compilation of molecular sequence datasets and our design of the phylogenetic analysis exhibit some major differences compared to earlier studies on the phylogeny of Holometabola. Specifically, i) we used a massive amount of data generated with Illumina Next Generation Sequencing (Table 1). ii) We ensured decisiveness of our datasets by specifically designing datasets for each of our seven research questions (Table 2) (see [22]). Decisiveness means that all genes included in a dataset are covered by at least one representative of all taxonomic groups that are relevant for the specific phylogenetic relationship under study. Accordingly, each dataset has a coverage of 100% in terms of presence of genes, with respect to the relevant taxonomic groups. By ensuring decisiveness, we alleviate the potentially misleading effects of missing data. Missing data can lead to inference of highly supported but wrong topologies (see [22]). iii) We performed FcLM [23] for each of our seven datasets (Table 3). We re-implemented FcLM in RAxML to cope with these large-scale data matrices and complemented the method by newly-written scripts that map respective results into 2D simplex graphs. Bootstrap support in phylogenetic trees alone is of limited conclusiveness in analyses of very large datasets [22,34]. FcLM is a method to identify possible support for alternative topologies in a dataset, *i.e.*, a method to display incongruent signal that might not be observable in phylogenetic trees. This study is the first to apply FcLM to large phylogenomic supermatrices. Finally, iv) we checked all datasets for rogue taxa. Rogue taxa are taxa that assume multiple phylogenetic positions in a set of bootstrap trees. They decrease resolution and/or support, for example, when building bootstrap consensus trees. Removing rogues may produce a more informative bootstrap consensus tree [35,36] (see Additional file 4, Chapter 4). All our datasets were free of rogues.

With a compilation of datasets as presented here (*i.e.*, by extracting the maximum number of genes that can contribute to resolving the phylogenetic relationship in question) we also ensured that inferred topologies were not based on an arbitrary selection of genes with respect to their inherent phylogenetic signal. Dell’Ampio et al. [22] showed that the selection of genes – if not driven by considerations concerning decisiveness of a dataset – can generate topologically different trees that may nonetheless all exhibit high support. Furthermore, Simon et al. [37,38] showed that genes involved in different biological pathways can support different topologies for a specific phylogenetic relationship. It can therefore be concluded that phylogenetic trees inferred from studying only a set of few to several genes are easily biased and thus might not reflect the correct species tree. While the currently best approach to address this problem is to include the maximum feasible amount of potentially informative data, we will have to further disentangle the contributing factors of topological incongruences in datasets (see also [22]).

Phylogenetic studies exclusively based on morphology (*e.g.*, [15,24]) also yielded problematic groupings in some cases. The authors addressed and discussed apparent artifacts that were mainly caused by parallel reductions in character complexes (*e.g.*, the flight apparatus). However, the problems turned out as intractable given the data and analytical procedures at hand [15]. With our molecular datasets we were able to provide reliable solutions for most interordinal phylogenetic relationships within Holometabola (Figure 1, and above). For tracing evolutionary changes on the phenotypic level we used the most extensive morphological dataset presently available, including 356 characters of representatives of all holometabolan orders and of carefully selected outgroup taxa [15]. The characters were mapped onto the transcriptome-based phylogeny in a formal approach (see Methods section for details). This allowed us to trace and re-interpret evolutionary changes of numerous characters and to conduct parsimony-based groundplan reconstructions for all clades of the tree (see “The evolution within Holometabola” below).

### The evolution within Holometabola

#### Larvae and development

**Figure 2:**
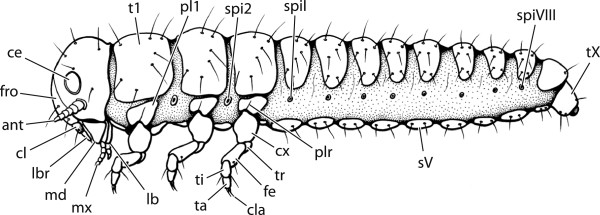
**Illustration of reconstructed groundplan larva of Holometabola.** The putative groundplan larva was orthognathous, and equipped with simplified but distinctly developed compound eyes, and well developed thoracic legs. Abdominal prolegs and cerci were absent. For a list of larval and adult groundplan characters of Holometabola, see Table 4. ce: compound eye. fro: frons. ant: antenna. cl: clypeus. lbr: labrum. md: mandible. mx: maxille. lb: labium. t1: tergite of first thoracic segment. pl1: pleurite of first thoracic segment. spi2: spiracle of second thoracic segment. plr: pleural ridge. cx: coxa. tr: trochanter. fe: femur. tib: tibia. ta: tarsus. cla: claw. spiI: spiracle of first abdominal segment. sV: sternite of fifth abdominal segment. spiVIII: spiracle of eighth abdominal segment. tX: tergite of tenth abdominal segment.

Our phylogenetic results suggest that the ancestral larva of Holometabola was terrestrial, orthognathous, equipped with moderately simplified but distinctly developed compound eyes, and well developed thoracic legs. Abdominal prolegs and cerci were absent (Figure 2). The muscle system was generally well developed. Distinct simplifications of the antennae and labial endite lobes and associated muscles are larval autapomorphies of Holometabola. The orthognathous head in the groundplan suggests that the earliest holometabolan larvae were feeding externally on plant material or fungi and not burrowing in substrate or penetrating narrow crevices (*e.g.*, under bark).

The ancestral aparaglossatan larva was likely prognathous and equipped with stemmata. Whether these larvae were of the agile campodeid type, like the larvae of many beetles (*e.g*., Adephaga, Myxophaga [*partim*], Staphylinoidea), Strepsiptera (first instar), Neuropterida, and some groups of Trichoptera (*e.g*., Rhyacophilidae), remains unclear. It is conceivable that this larval type is an apomorphic condition characterizing Neuropteroidea, with parallel evolution in Trichoptera. Prognathism is often linked with carnivorous feeding habits (Neuropterida, Adephaga, and some polyphagan subgroups), but can also be related with penetrating narrow crevices or burrowing in substrates, as it is the case in the wood-associated larvae of Archostemata (Coleoptera), but also in early lepidopteran lineages (*e.g*., [1]). Thus, it is unclear whether or not the ancestral aparaglossatan larvae were predaceous. Larvae of Mecopterida display some simplifications (tentorium and antennal segments), and a distinct trend towards reductions characterizes antliophoran larvae, especially those of Siphonaptera and Diptera. Both have entirely lost their thoracic legs (distinctly shortened in Mecoptera) and are characterized by simplifications of cephalic structures, especially of the muscle system [39]. This reflects the widespread larval life history in Antliophora, with larvae living in the upper soil layer, leaf litter, moist substrates, or different water bodies, feeding mainly on soft substrates or small particles. The important question whether ancestral antliophoran larvae were terrestrial (Lepidoptera, Mecoptera, Siphonaptera, Mecoptera excl. Nannochoristidae, Diptera *partim*) or aquatic (Trichoptera, Nannochoristidae, Diptera *partim*) remains ambiguous.

Our phylogenetic results clearly indicate that a typical holometabolous development with larvae completely lacking external wing buds (“endopterygote insects”) and also lacking cerci belongs to the groundplan of Holometabola (see also [1,17]). The conditions characterizing strepsipteran primary larvae (abdominal segment XI and cerci present) and secondary larvae (external wing buds recognizable as external convexities) are apparently the result of reversals, like the early appearance of the prospective compound eyes (see [17]). Largely immobilized pupae with immobilized mandibles (pupa adectica) have almost certainly evolved several times independently. It appears likely that a mobile pupa with movable mandibles as it is characteristic for Raphidioptera is ancestral for Holometabola even though this is not confirmed by a formal character analysis.

#### Adults and egg deposition

The ancestral holometabolan adult apparently differed only slightly from the neopteran groundplan (Neoptera: all winged insects except Odonata and Ephemeroptera). Cephalic structures, the entire muscle system, the flight apparatus, and abdominal structures appear largely unmodified [15,23,39,40]. The most profound apomorphies in adult holometabolan insects are related to the invagination of the pterothoracic sternites (*e.g.*, closely adjacent meso- and metacoxae) [24]. Our data do not lead to a reliable assessment of ancestral feeding habits of holometabolan adults, but it is apparent that feeding in the adult stage played a minor role compared to feeding in the larval stages. Exceptions to this rule are for instance predaceous beetles (*e.g*., Dytiscidae and Carabidae) with a very rapid postembryonic development and long-lived adults.

Distinct morphological character transformations characterize the rise of Aparaglossata: the reduction of the labial endite lobes (paraglossae), including muscles, the distinct modification of the orthopteroid ovipositor, and possibly the reduced number of Malpighian tubules (also in Acercaria (true bugs, psocopterans, lice, and relatives)) [15,41]. Our results do not allow for an unambiguous reconstruction of the ancestral condition of the flight apparatus for Holometabola and Aparaglossata. It appears plausible that approximately equally sized pterothoracic segments (as in Neuropterida, early lepidopteran lineages, and Mecoptera) are plesiomorphic for Aparaglossata, but the reconstruction of the ancestral state of this character in the formal analysis remained ambiguous. As pointed out above, the question whether or not Coleopterida is a monophylic group is not completely settled. However, it appears plausible to assume that posteromotorism evolved only once in a common ancestor of Strepsiptera and Coleoptera, with a suite of related features, such as the size reduction of the mesothorax, a distinct reduction of the mesothoracic muscle system [42], and an increased size of the metathorax. A distinct anteromotorism as it is present in Hymenoptera, Trichoptera, “higher” Lepidoptera, and Diptera is possibly ancestral in Holometabola, but it is conceivable that this condition has evolved (secondarily?, see above) several times independently (*e.g.*, almost equally sized pterothoracic segments in non-glossatan Lepidoptera).

Wing coupling mechanisms have apparently evolved independently in Hymenoptera (hamuli as an autapomorphy of the order, see Additional file 4, Chapter 5), Trichoptera, Lepidoptera, and some families of Neuroptera (different mechanisms occur in these orders).

The primary mode of egg deposition in Holometabola was very likely endophytic, as it can be assumed for the groundplan of Hymenoptera (“Symphyta”). This mode of egg deposition is arguably maintained in the groundplan of Neuropteroidea. Raphidioptera have a modified, elongated ovipositor which they use to deposit eggs under bark or into ground litter. This resembles egg deposition as assumed for the groundplan of Holometabola and Hymenoptera; however, it might also be a derived character. The complete or nearly complete reduction of elements of the primary ovipositor is a characteristic of Mecopterida and obviously related with superficial egg-deposition or oviposition in soft substrates. Our results mostly confirm an evolutionary scenario for the female postabdomen and egg-deposition as outlined in detail in Hünefeld et al. [41].

## Conclusions

**Figure 3:**
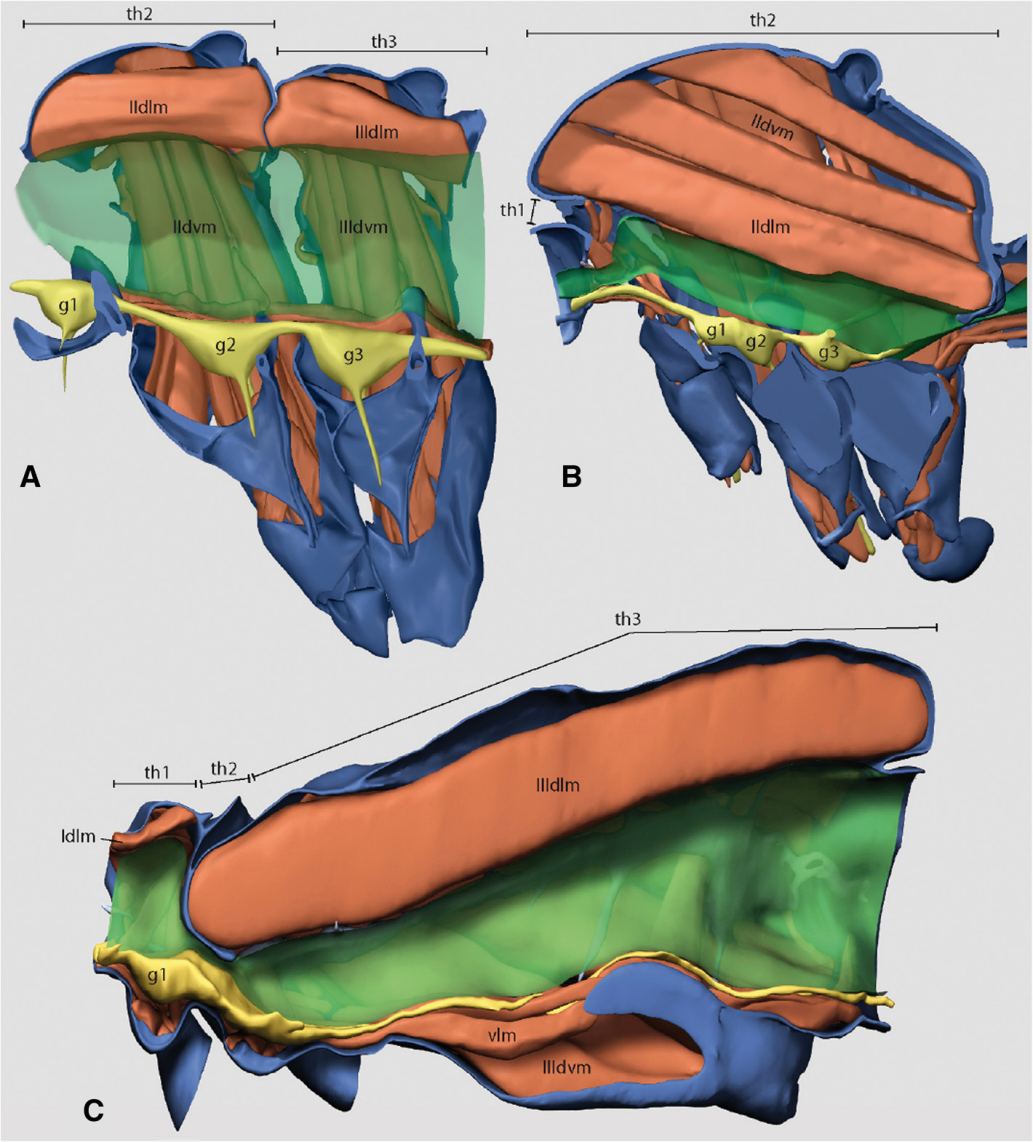
**Three holometabolan adult thorax states. A)** A thorax with approximately equally sized pterothoracic segments is possibly ancestral for Aparaglossata (Figure shows thorax of *Nannochorista neotropica* (Mecoptera, Nannochoristidae); prothorax not shown.). **B)** shows a thorax of taxa with anteromotorism, *i.e.*, flight with mainly the fore wings (*e.g.*, Hymenoptera, Trichoptera, “higher” Lepidoptera, and Diptera; figure shows *Ptychoptera* sp. (Diptera, Ptychopteridae)). This state is possibly ancestral for Holometabola. However, the reconstruction of the ancestral state of this character in the formal analysis remained ambiguous for Holometabola and Aparaglossata. **C)** shows a thorax of taxa with posteromotorism, *i.e.*, flight with the hind wings (Coleoptera and Strepsiptera; figure shows *Mengenilla moldrzyki* (Strepsiptera, Mengenillidae)). red: muscles. blue: sceleton. green: gut. yellow: nerves. Numerals refer to thoracic segments. th: thorax segment. g: ganglion. dlm: dorsal longitudinal muscle. dvm: dorso-ventral muscle. vlm: ventral longitudinal muscle (not visible in **A** and **B)**. A 3D version of this figure can be found as Additional file 5 (Click on image to activate animation).

Our transcriptome-based phylogenetic results allowed a reconstruction of transformations of morphological characters of larvae and adults. To summarize our findings, we show a hypothesized ancestral holometabolan larva in Figure 2, and a selection of adult and larval groundplan features in Table 4 (see Additional file 4, Chapter 5 for a full list). The ancestral state of the adult thorax remained ambiguous. Three main holometabolan types are shown in Figure 3 (and in Additional file 5 as 3D pdf). A selection of apomorphic features of the major subgroups of Holometabola whose phylogenetic origins have now been elucidated is presented in Table 4 (see Additional file 4, Chapter 5 for a full list).

For the first time in insect systematics a scenario for transformations on the phenotypic level is based on a strictly formal procedure, using a well-documented comprehensive morphological data-set in combination with analyses of phylogenomic data. Our combined approach may lead to a new level of reciprocal enlightenment between researchers with a main focus on morphology and molecular data, respectively, and eventually to new and well-founded insights into the evolution of Hexapoda and other groups of organisms.

## Methods

### Data acquisition

Our study included a total of 88 species: 71 holometabolan species, and 17 species belonging to different hemimetabolous lineages for outgroup comparison. Of these, we generated transcriptomic data *de novo* for 13 holometabolan species. From all remaining species, we used published transcriptomic data or the transcripts of the official gene set (OGS) if the genome of a species is already sequenced (see below).

The 13 holometabolan species (at least one representative of each order) with newly generated transcriptomic data are listed in Table 1 (for details see Additional file 6, Table S1). Extraction of RNA, cDNA library construction, library normalization, sequencing of 12.5 million paired end reads (~ 2.5 Gigabases raw reads per species) using the Illumina Technique (Hiseq 1000), and sequence processing (vector-clipping, trimming and soft-masking of raw reads, and assembly into contigs) were done by LGC Genomics, Berlin, Germany (see Additional file 4, Chapter 1, and Additional files 6 and 7: Tables S1 and S2 for details). All raw nucleotide sequence reads are deposited at the NCBI Sequence Read Archive (SRA). The corresponding nucleotide assemblies have been deposited at the NCBI's Transcriptome Sequences Database (TSA) (Umbrella project ID PRJNA176423). For further details and accession numbers, please refer to Additional file 4, Chapter 1, and Additional file 7: Table S2.

Nucleotide sequence assemblies of published transcriptome data were obtained from the Deep Metazoan Phylogeny (DMP) database (http://www.deep-phylogeny.org/), NCBI's Transcriptome Sequences Database (TSA) and from various web sources of species whose official gene set was available. We only used species with more than 3,000 available contigs (status: November 2012) (Additional file 8: Table S3).

### Orthology assignment

We mapped the transcripts to a set of 1,343 ortholog groups (OGs), *i.e.*, a set of genes that have been identified as single-copy orthologs in 14 reference species (13 insects, 1 crustacean) in OrthoDB 4 (http://cegg.unige.ch/orthodb4/) (see Additional file 9: Table S4 for reference species, and Additional file 10: Table S5 for included orthologs; for details on the design of the ortholog reference set see Additional file 4, Chapter 2). Orthology of transcripts was assigned using HaMStRad, a modified version of HaMStR v.8 [43] (see Additional file 4, Chapter 2 for details on modifications). The modified program files are available at https://github.com/mptrsen/HaMStRad (Status: March 2013). HaMStRad maps transcripts to a set of OGs using hidden Markov models and the best reciprocal hit criterion. We ran HaMStRad with the following settings: (i) the E-value cut-off for the pHMM search was 1e-5, (ii) the reciprocity criterion was considered fulfilled if the candidate OG was found as best hit in at least one of the 14 reference species during the reciprocal best hit search (RBH) (−*relaxed* option), (iii) in case of multiple transcripts being assigned to a given OG, the best set of non-overlapping transcripts was chosen while non-overlapping transcripts are automatically concatenated (−*representative* option). Transcripts that were assigned to more than one OG were removed from the dataset using Perl scripts (available upon request) (redundancy check). Furthermore, we removed terminal stop codons and masked internal stop codons with ‘X’.

### Multiple amino acid sequence alignment, refinement, and masking

We aligned all OGs separately at the amino acid level using MAFFT L-INS-i [44] v6.951. Then we checked for misaligned sequences (henceforth called “outliers“) in multiple amino acid sequence alignments (MSAs) of all OGs. This check was done with Perl scripts (available upon request) applying the following procedure: first, the maximal alignment length of a given multiple amino acid sequence alignment was recorded. Then, mean, median, and quartiles of BLOSUM62 distances of the amino acid sequences of all reference species were calculated. After that, the BLOSUM62 distance of each transcript to the sequence of its closest reference taxon (*i.e.*, the reference taxon found as best reciprocal hit) was calculated. Subsequently, it was checked whether this distance was below or above a cut-off value of 2.25 times the distance of the upper quartile to the mean of the BLOSUM62 distances among the reference species. Transcripts with a minimal BLOSUM62 distance to a reference species above the cut-off were classified as outliers, and also sequences with less than 20 overlapping sites to the corresponding sequence of the reference species. All outliers were extracted from the respective MSAs. Each outlier amino acid sequence was separately aligned to only the aligned orthologous sequences of the reference species, using the "--add" option in MAFFT L-INS-i. The refined outlier amino acid sequences were reintegrated into the respective MSA using the alignment of the reference species as a backbone. The outlier check procedure as described above was repeated for each MSA. Sequences that were still classified as outliers were finally removed from the respective MSA (see Additional file 8: Table S3). Gap-only sites were also removed from the MSAs.

Ambiguously aligned sections were identified with a modified version of ALISCORE [45-47]; for modifications, see [47]). We applied the default sliding window size, the maximal number of pairwise comparisons (−*r* option) and a special EST data scoring (−*e* option). Identified ambiguously aligned sections were removed (“masked“) from the MSAs with ALICUT v.2.0 ([48], http://www.museumkoenig.de/web/ZFMK_Mitarbeiter/KckPatrick/Software/AliCUT/Download/index.de.html) (see Additional file 11: Table S6).

### Design of seven specific decisive datasets addressing particular phylogenetic relationships

We call a dataset phylogenetically decisive if all included OGs contain at least one sequence of a representative of each taxonomic group of interest. To compile decisive datasets, we selected four taxonomic groups of interest for each of our seven phylogenetic questions (Table 2 and Table 3). All species relevant for a specific question were assigned to one of the four groups (also called “clusters“, see below; see also Additional file 12). The monophyly of each group of species is assumed. All OGs that contained at least one sequence of a representative of each group were extracted with Perl scripts (available upon request) and concatenated into seven supermatrices that constitute the seven decisive datasets. The taxa that are not relevant for answering the respective question were removed (see also Additional file 13: Table S7). The amount and distribution of missing data in each dataset was visualized with *mare* v. 0.1.2-rc ([49], http://mare.zfmk.de) (Additional file 1: Figures S1-S7).

### Phylogenetic analyses

For each of the seven datasets, we performed phylogenetic tree reconstruction with the maximum likelihood (ML) optimality criterion and Four-cluster Likelihood Mapping (FcLM) at the amino acid level. We refrained from calculating the Relative Composition Variability (RCV, see [50]) among the sequences in a dataset to select an optimal data subset (*e.g.*, first, second, and third codon positions of nucleotide sequence dataset, and amino acid sequence dataset) because the statistics is not independent of sequence length, number of sequences, and frequency of symbols. This renders a comparison of RCV between datasets with a different number of symbols and different lengths inappropriate.

For maximum likelihood tree inference, the smaller and larger datasets were treated in slightly different ways because of RAM limitations. For analyzing our small datasets (datasets 3, 4, 5, 7), we conducted one tree-search per dataset to determine the best fitting model, using the −*AUTO* function implemented in RAxML-Light [51] v. 1.0.9., under the GAMMA model of rate heterogeneity [52] using the median for the discrete GAMMA approximation. Then, ML trees for the small datasets were inferred applying the −*f a* command line option in RAxML [53], v.7.3.1, HYBRID [54,55] with the CAT model of rate heterogeneity [53], the best-scoring amino acid substitution matrix, and empirical amino acid frequencies (PROTCAT, bestMODEL, F option). The final tree-searches were conducted under the GAMMA model of rate heterogeneity, again using the median for the discrete approximation. For analyzing our larger datasets (1, 2, and 6), we used RAxML-Light v. 1.0.9 to determine the best-scoring protein substitution model and for subsequent tree inferences. Based on randomized topologies of starting trees, we conducted 50 tree-searches with the CAT model of rate heterogeneity (PROTCATAUTOF) and estimated the best-scoring model using empirical frequencies (+ F) for each tree-search. We subsequently estimated the best final GAMMA likelihood and additional parameters under the GAMMA model using the median for the discrete approximation. For all datasets, the best-scoring amino acid model was the LG model [56].

We assessed statistical support for each node from bootstrap replicates. Bootstrap analyses were performed with the rapid bootstrap algorithm [53], using bootstopping criteria ([57], command line option: −# autoMRE -B 0.01). For analyzing the small datasets, the search for the best tree and the bootstrap analyses were performed in one single step (−*f a* option). For analyzing the large datasets, bootstrap analyses were performed separately and the bootstrap support was plotted on the respective best tree.

All ML analyses were conducted on Linux clusters at the Cologne High Efficient Operating Platform for Science (CHEOPS), Regionales Rechenzentrum Köln (RRZK) (http://rrzk.uni-koeln.de/cheops.html).

After tree inference, we scrutinized our trees for rogue taxa ([36,58], see Additional file 4, Chapter 4).

Trees were edited with Treegraph 2.0 [59], and rooted with respective outgroups (see Additional file 2: Figures S8-S15). Supermatrices (*i.e.*, datasets) are deposited at labarchives repository, DOI10.6070/H4G73BMJ, https://mynotebook.labarchives.com/share/ubulin/MC4wfDIzNDAzLzAvVHJlZU5vZGUvMjA0NzAzNzkzMHwwLjA.

### Four-cluster Likelihood Mapping (FcLM)

We used FcLM proposed by Strimmer and von Haeseler [23] as an alternative method for analyzing single phylogenetic splits. In each decisive dataset, all included species were binned into four clusters that correspond to the taxonomic groups that are relevant for the respective phylogenetic relationship (see above, Table 2, and Additional file 12). The phylogenetic relationships between these four clusters represent the phylogenetic question of interest. In one case (dataset 6), we defined two different sets of clusters because two phylogenetic hypotheses had to be tested. For each dataset, we calculated the log-likelihood values of all non-redundant quartets drawn from the predefined species groups (“clusters”) (see Additional file 4, Chapter 3). We implemented this in RAxML (as of v. 7.3) to be able to handle large-scale datasets. Calculation of log-likelihood values was performed using the GAMMA model of rate heterogeneity and empirical base frequencies with RAxML 7.3.1 (PTHREADS) on the MESCA System of the HPC Linux Cluster CHEOPS, RRZK, University of Cologne. We developed an additional tool written in Perl to map the support values of the RAxML analyses for each quartet onto 2D simplex graphs (available upon request). Results from the analysis of all seven datasets were plotted on the main tree (Figure 1). For the final phylogenetic inference, we compared support inferred from FcLM with ML bootstrap support.

### Additional partitioned ML tree and FcLM analyses of dataset 5

We repeated ML tree reconstruction and FcLM based on partitioned analyses for dataset 5 to identify possible sources for incongruence between results of tree reconstruction and FcLM in this specific case. For the partitioned ML tree reconstruction (with 972 partitions), we followed the procedure applied on the large datasets (see above), but using ExaML (version 4.1 [2013-06-19]) instead of RAxML-Light, with the PSR model of rate heterogeneity (equal to CAT in RAxML-Light). We subsequently estimated the optimal parameters and the log-likelihood using the GAMMA model of rate heterogeneity. We performed 50 tree searches and choose the one with the best log-likelihood as best tree (Additional file 2: Figure S15). For partitioned FcLM analysis, we used the respective best models for each partition, selected during the preceding ML tree search (–*AUTO* option in RAxML), as input (Additional file 14: Table S8). For calculating the log-likelihood support for each drawn quartet, we used again the GAMMA model of rate heterogeneity and empirical base frequencies in RAxML 7.7.2 (PTHREADS). Results were again mapped onto a 2D simplex graph (Additional file 3: Figure S25).

### Reconstruction of character evolution and groundplans

Morphological characters of immatures and adults were mapped onto the reconstructed tree using Mesquite ([33], http://mesquiteproject.org). As input, we used the datamatrix of morphological characters published by Beutel et al. [15] and the interordinal topology of the transcriptome-based phylogeny inferred from dataset 1, which represents the complete molecular datamatrix (Figure 1). The taxon sampling at the species level is not congruent between Beutel et al. [15] and the present study. However, all orders are covered in both studies, and only evolutionary transformations between orders or supraordinal taxa are considered here. To reconstruct the character evolution and groundplan features at each node, we used the “Trace Character History” option and performed maximum parsimony reconstructions of groundplans (select “Parsimony Ancestral States”) for categorical characters under unordered states assumption.

### Availability of supporting data

The datasets supporting the results of this article are available in the labarchives repository, DOI10.6070/H4G73BMJ, https://mynotebook.labarchives.com/share/ubulin/MC4wfDIzNDAzLzAvVHJlZU5vZGUvMjA0NzAzNzkzMHwwLjA.

## Competing interests

The authors declare that they have no competing interests.

## Authors’ contributions

The study was conceived by BM, KM, KMK, UA, HA, RSP and RGB. MP and KM compiled the ortholog reference set. MP wrote HaMStRad. JW, MP, TZ, ON, CM, and BM wrote all necessary Perl scripts. AD conducted VecScreen analyses and did the submission of data. KM conducted all molecular data analyses except alignment refinement (done by CM) and rogue taxon analyses (done by AJA). AS re-implemented and parallelized the likelihood calculations on quartets in RAxML. Analyses of morphological data were done by RGB and FF. The manuscript was written by RSP, KM, RGB and BM with useful comments and revisions from all other authors. All authors read and approved the final manuscript.

## Supplementary Material

Additional file 1: Figures S1-S7 Presence and absence of genes in datasets 1 to 7. Files visualize the data matrices of datasets 1 to 7, in terms of gene coverage (Figure S1: dataset 1 to Figure S7: dataset 7). Grey dot: gene present. White dot: gene absent. The data matrices were visualized with *mare*[49]. Click here for file

Additional file 2: Figures S8-S15 Full phylogenetic trees, inferred from ML analyses of datasets 1 to 7. Files show full phylogenetic trees, inferred from maximum likelihood (ML) tree reconstructions of datasets 1 to 7 (Figure S8: dataset 1 to Figure S14: dataset 7; Figure S15: best tree of the additional partitioned analysis of dataset 5). Branches with <50% bootstrap support are shown as unresolved. Species for which new transcriptome data were generated in this study are in bold print. For details of phylogenetic tree reconstruction, see Methods section of main text. Click here for file

Additional file 3: Figures S16-S25 Results of the Four-cluster Likelihood Mapping (FcLM) as 2D simplex graphs. Figure S16. Exemplary 2D simplex graph based on the Four-cluster Likelihood Mapping (FcLM). For explanations see Additional file 4, Chapter 3. Figures S17-S25. 2D simplex graphs showing results of the Four-cluster Likelihood Mapping (FcLM) of datasets 1 to 7 (Figure S17: dataset 1 to Figure S221: dataset 5; Figure S22 and S23: dataset 6a and 6b; Figure S24: dataset 7, Figure S25: additional partitioned analysis of dataset 5). Left: the support for each quartet is shown as a single dot mapped onto the 2D simplex graph. Right: proportion of quartets with predominant support for the respective topology is given. For details on methods, topologies T1, T2, and T3, and interpretation of results see Methods and Results section of the main text, Additional file 4, Chapter 3, and Figure S16. Click here for file

Additional file 4 **More details on methods and results.** The text gives more detailed information on methods (generation of new transcriptome data and retrieval of published data, orthology assignment, and Four-cluster Likelihood Mapping), and provides additional results (rogue taxa, morphological analyses). Click here for file

Additional file 5: Figure_3_3D Figure 3 of main text as 3D pdf. Click on image to activate animation. Click here for file

Additional file 6: Table S1 Species for which new transcriptome data were generated, with collecting and preservation information. This table gives all available metadata for the species for which new transcriptome data were generated in this study, including, for example, collecting information, species identifying person, sex and stage, preservation details. Click here for file

Additional file 7: Table S2 Statistics of newly generated transcriptome data. This table gives statistics of the generated data, *e.g.,* number of raw reads, number of contigs after assembly, length of contigs, and accession numbers at NCBI GenBank. All data can be found at NCBI Umbrella BioProject ID: PRJNA176423 - Evolution of holometabolous insects; BioProject accession number: SRP015962. For details on linker clipping and quality trimming see Additional file 4, Chapter 1. Click here for file

Additional file 8: Table S3 All species included in this study, including previously published data. Listed are sources for download of data, results of orthology assignment, and results of subsequent quality assessment steps (see Methods section of main text for details). Capitalized species: whole genome sequence and an official gene set are available. Species marked with an asterisk were used as reference species in the ortholog reference set, see Additional file 4, Chapter 2 for details. Click here for file

Additional file 9: Table S4 Reference species used in the ortholog reference set. Table lists the species that were used during compilation of the ortholog reference set, see Additional file 4, Chapter 2 for details, and information on download source and date. *Daphnia pulex* was used as reference species but not included in the taxon sampling. Click here for file

Additional file 10: Table S5 List of 1,343 ortholog groups (OGs) included in the ortholog reference set. Table lists all OGs analyzed in this study, with OG ID, Uniprot ID, and preliminary annotation. Annotation was retrieved from OrthoDB4, either using a consensus rule for OGs marked with an asterisk, or adopting the annotation of *Pediculus humanus;* 'x' indicates the complete removal of an annotation during the cleaning process (see Additional file 4, Chapter 2 for details). Click here for file

Additional file 11: Table S6 Proportion of excluded ambiguously aligned sites (%) for each ortholog group. In each ortholog group, alignment sections which were evaluated as ambiguous with ALISCORE were excluded prior to compilation of datasets 1 to 7, subsequent ML tree reconstruction and FcLM (see Methods section of main text for details). Click here for file

Additional file 12 **Species groups selected for the design of decisive datasets.** For design of our seven datasets, we selected four taxonomic groups each of which is relevant to address a phylogenetic relationship in question. Species were binned into these four groups. In this file, we list the species included in each group for each of our datasets. Click here for file

Additional file 13: Table S7 Number of ortholog groups (OGs) per species and dataset. Table lists how many OGs are covered by each species in the seven datasets that were analyzed in this study. Click here for file

Additional file 14: Table S8. Best scoring model of each partition in partitioned analyses of dataset 5. The table lists the selected model for each partition of dataset 5, using the AUTO option implemented in ExaML, applied in the additional partitioned analyses (ML tree reconstruction and FcLM). Click here for file
