# Supplementary figures and images for: The evolutionary history of holometabolous insects inferred from transcriptome-based phylogeny and comprehensive morphological data
Source: BMC Evol Biol. 2014 Mar 20;14:52. doi: 10.1186/1471-2148-14-52 (PMC4000048; doi:10.1186/1471-2148-14-52)

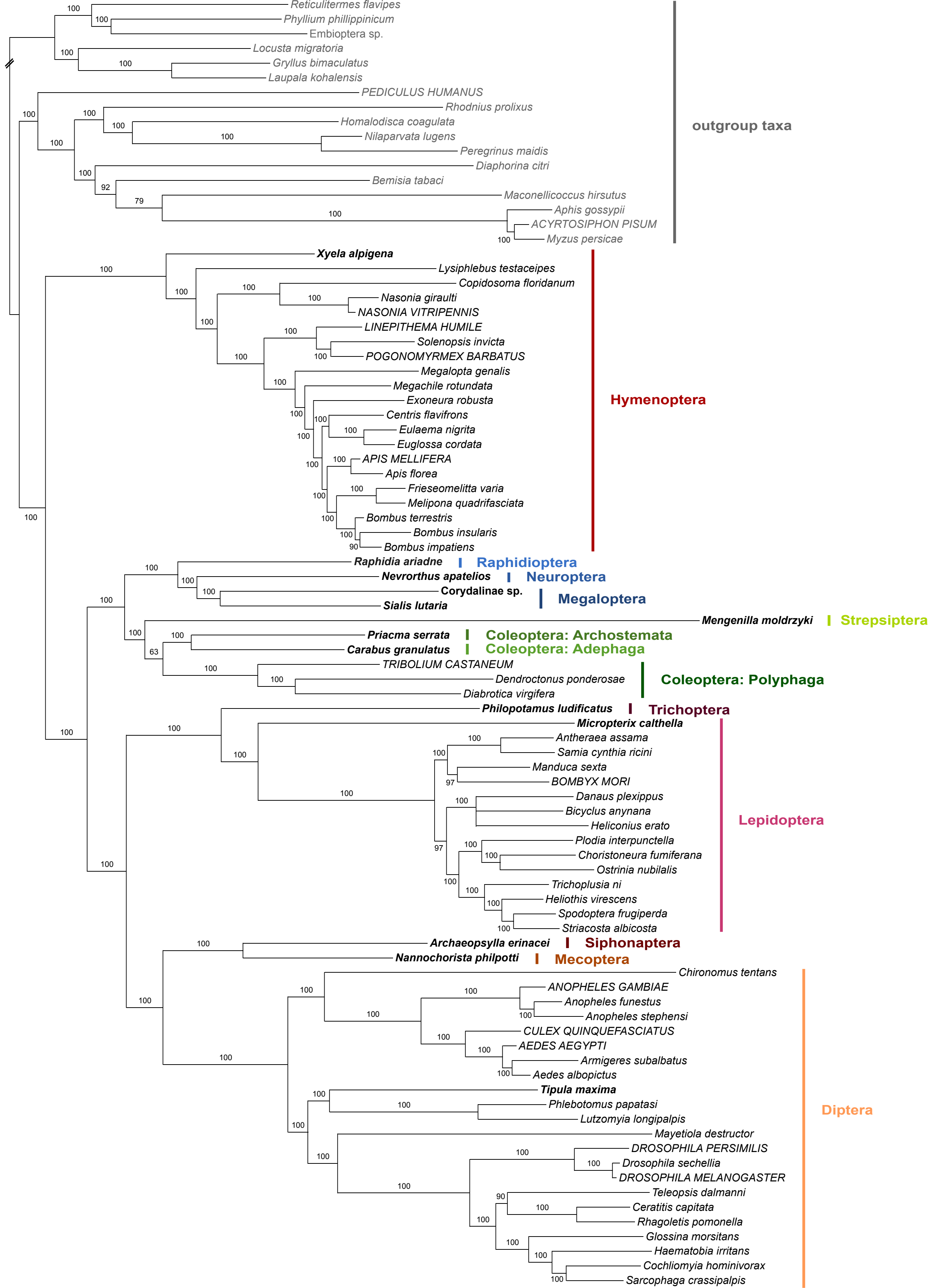

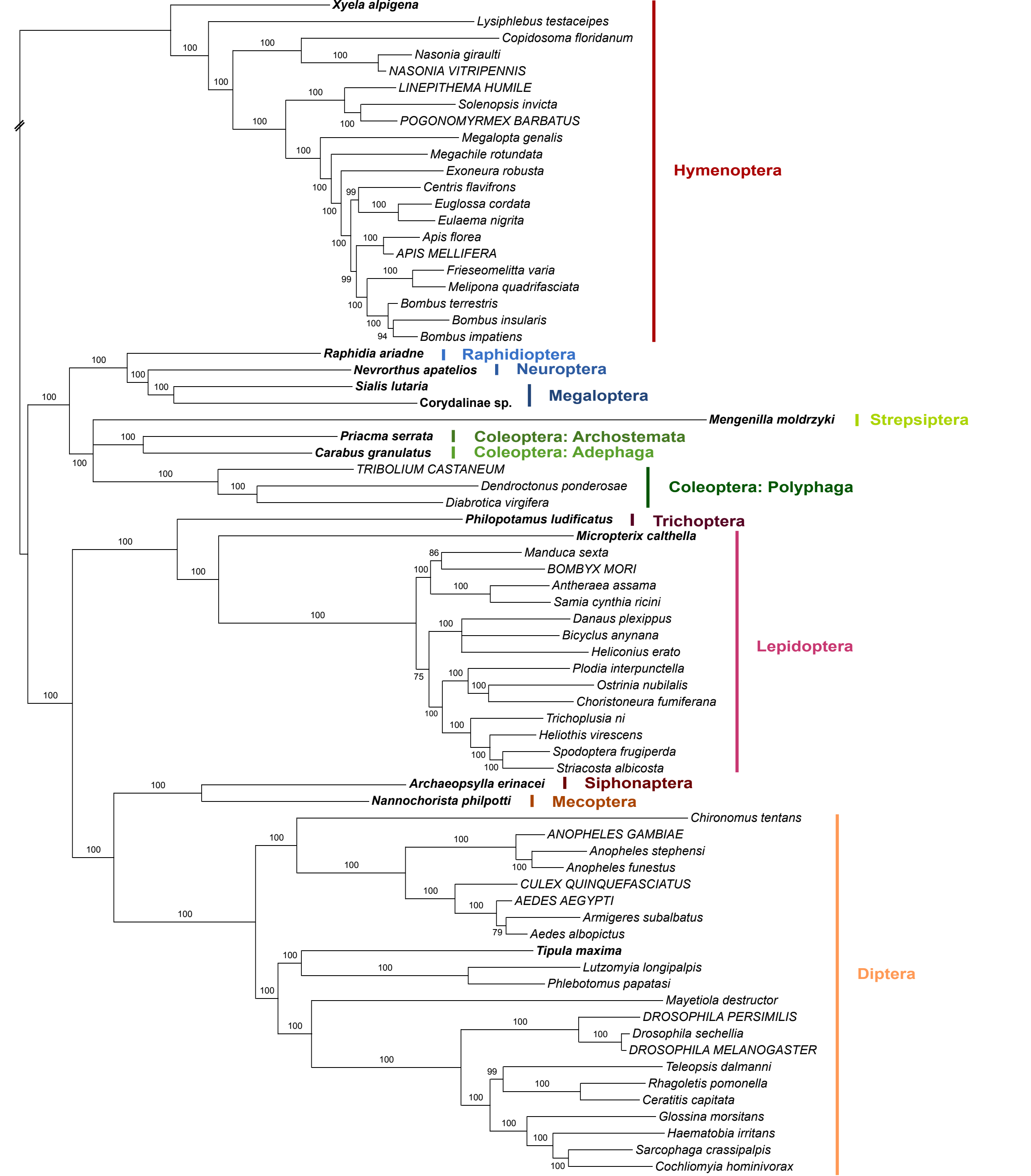

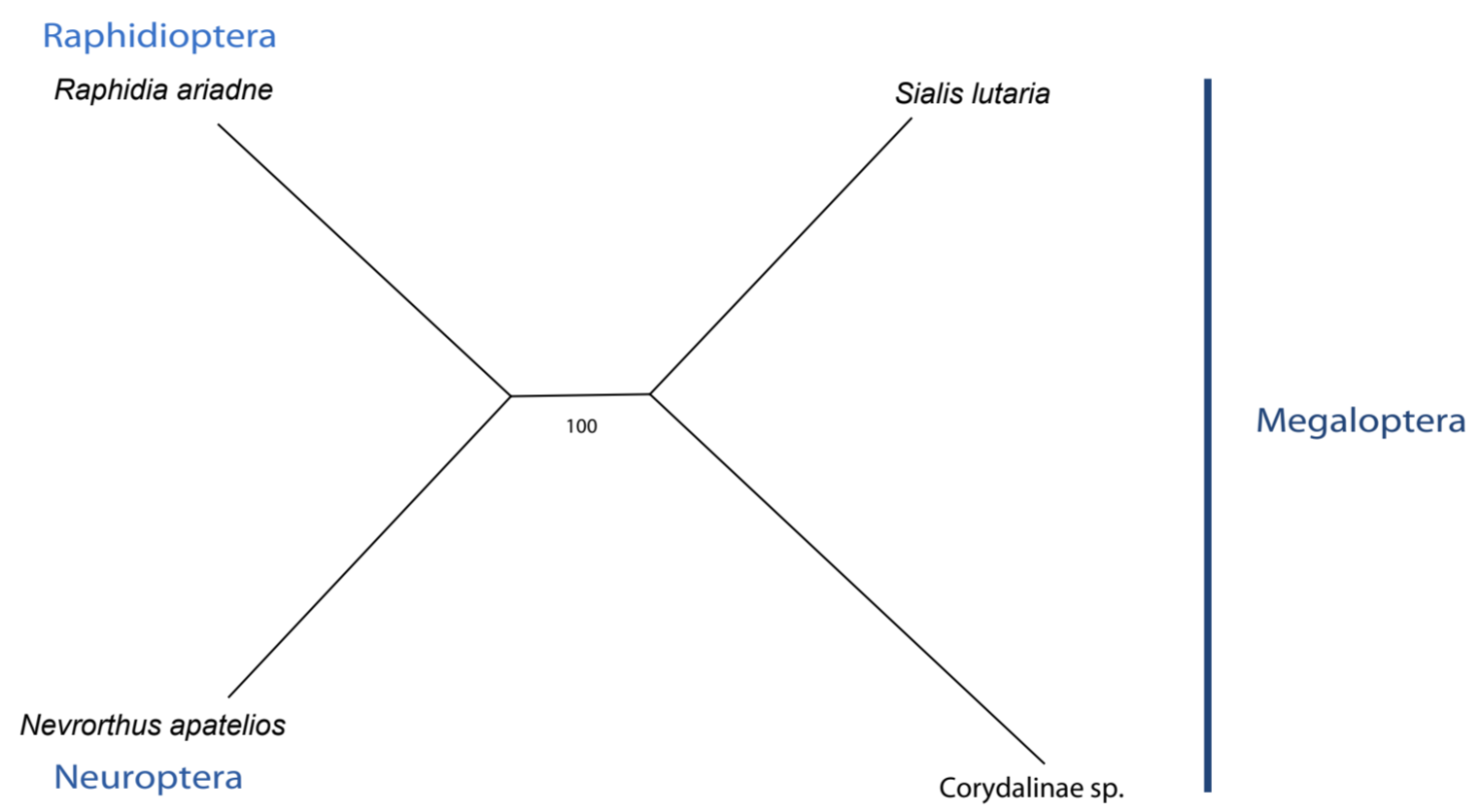

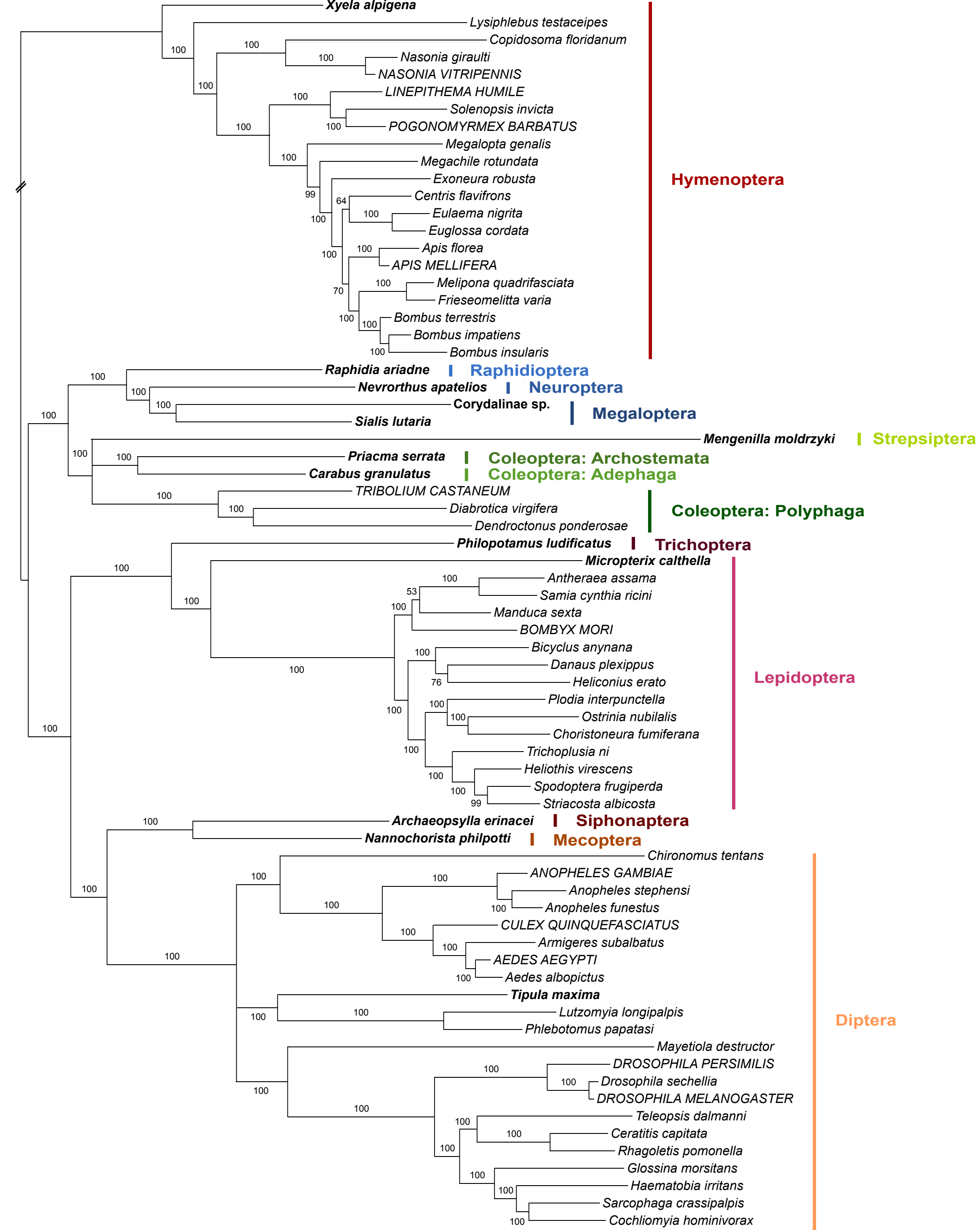

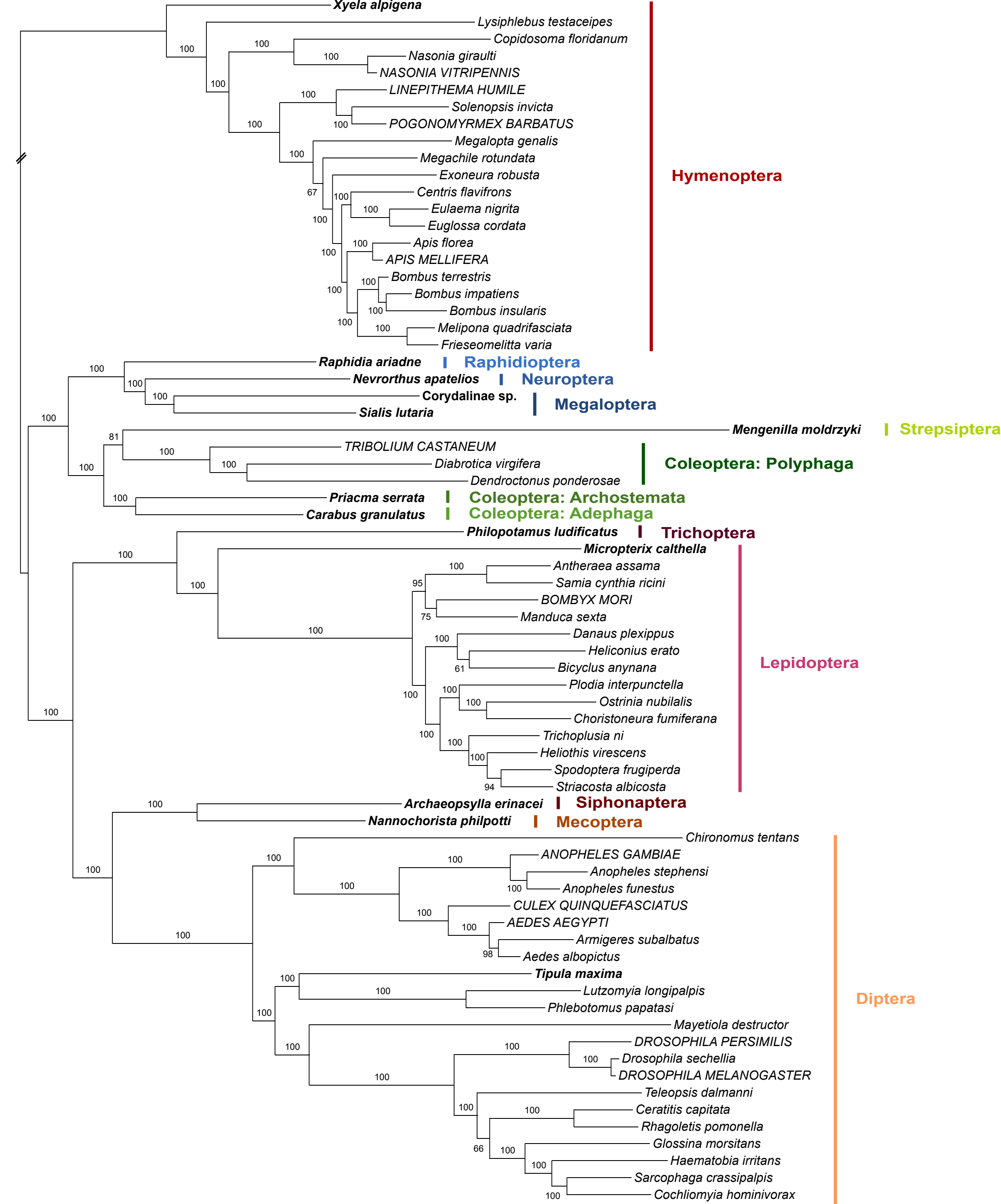

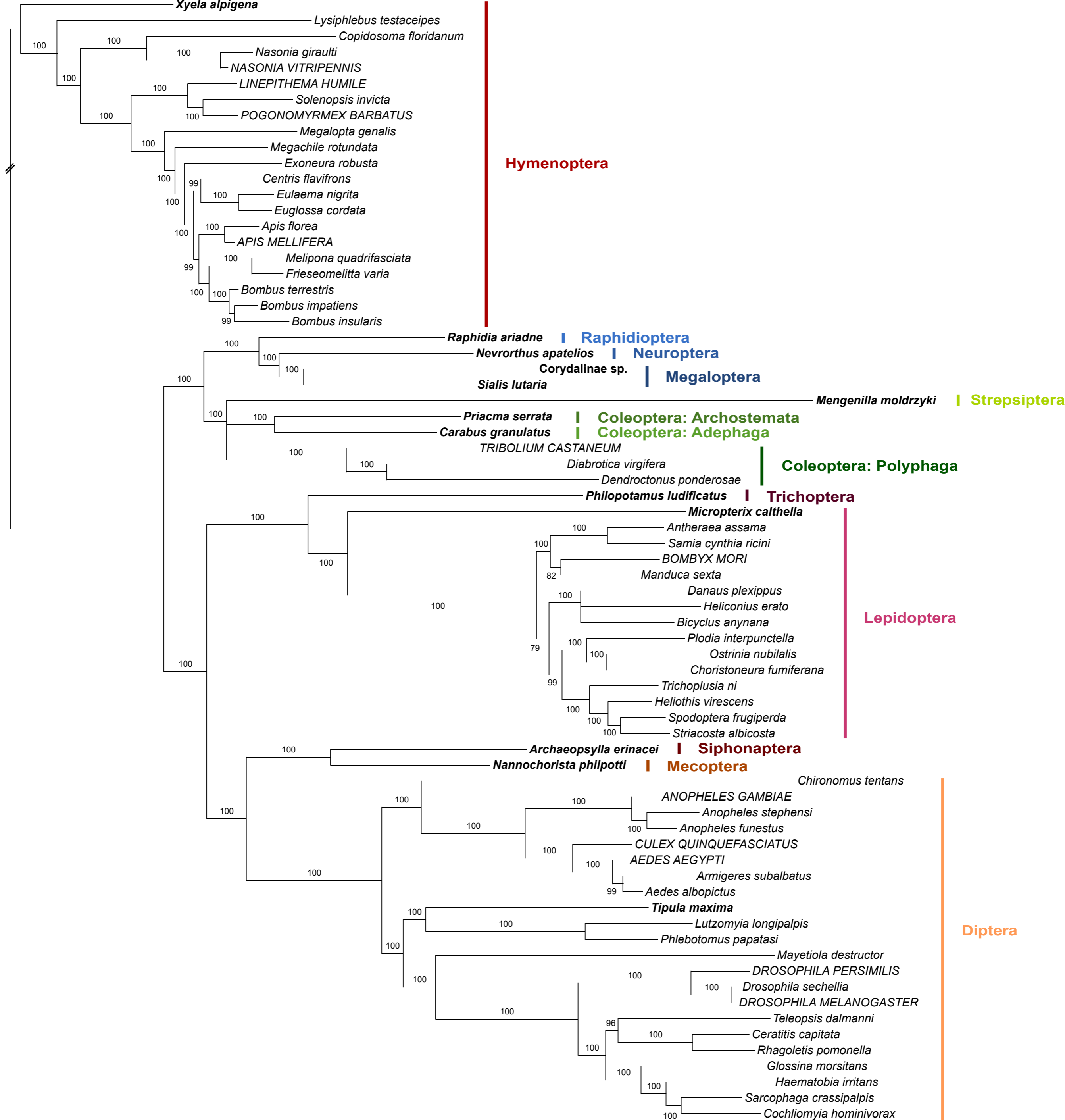

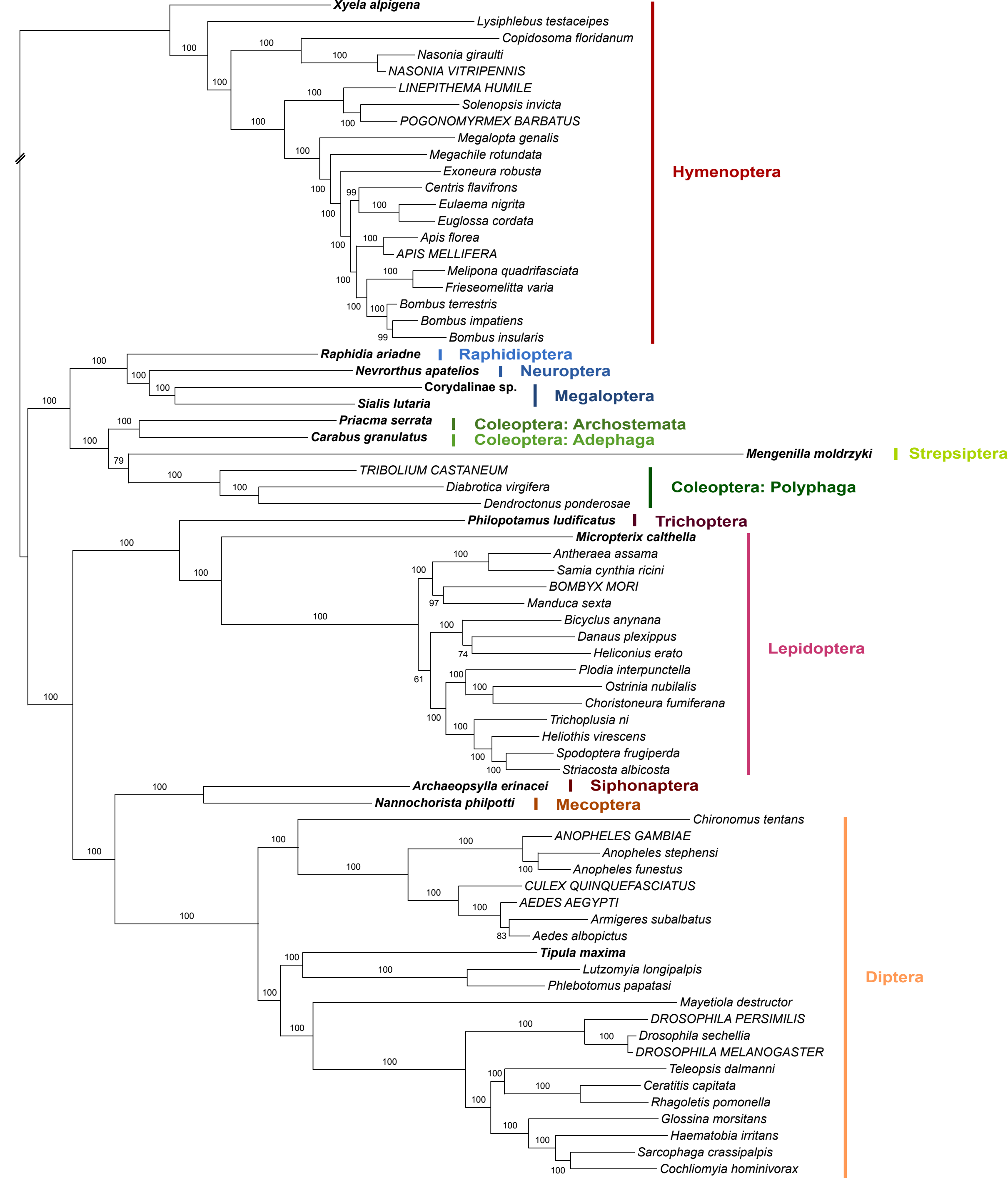

Dataset 5, best ML tree derived from partitioned analysis

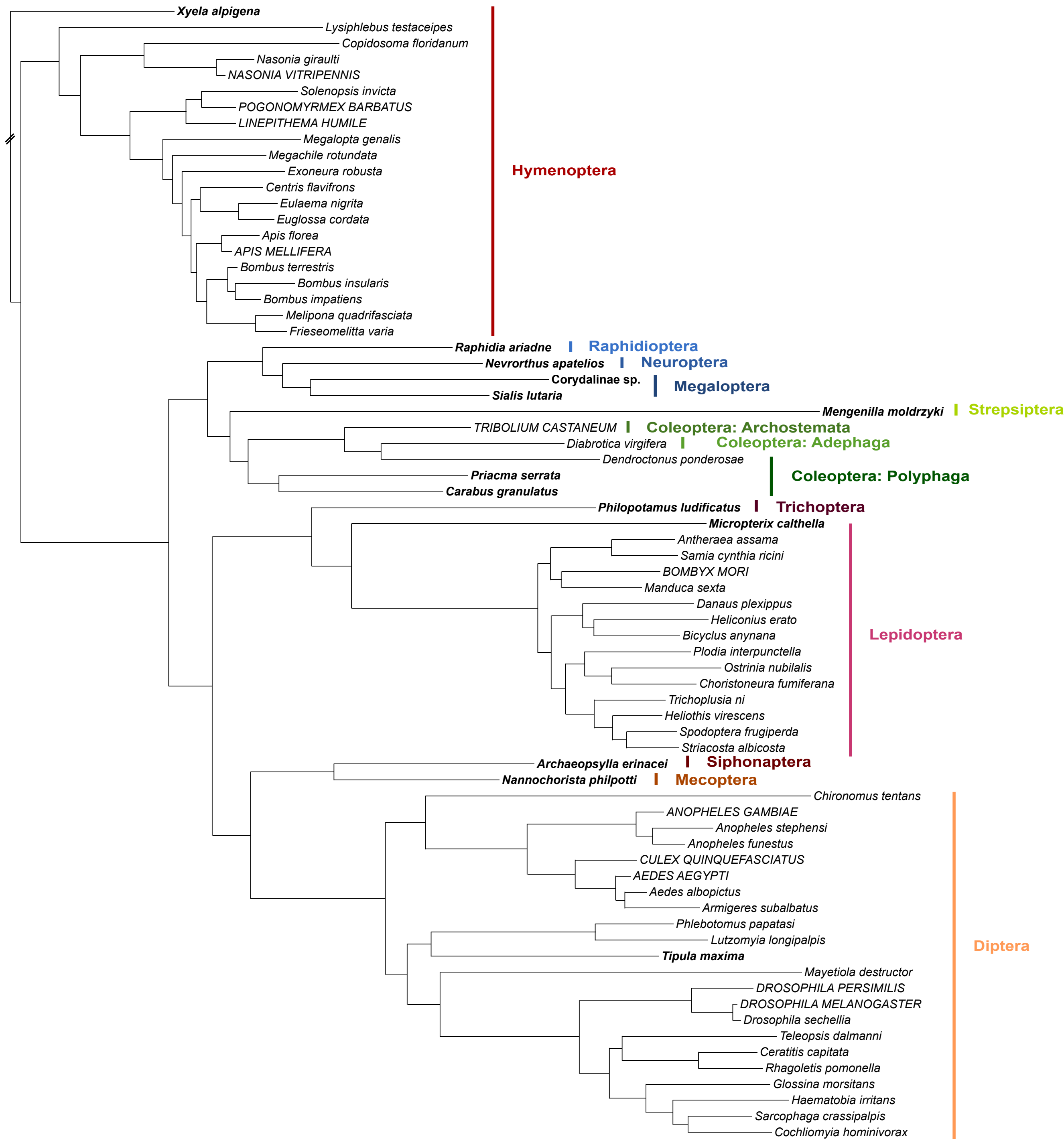

Supplement: Additional file 2: Figures S8-S15 — Full phylogenetic trees, inferred from ML analyses of datasets 1 to 7. Files show full phylogenetic trees, inferred from maximum likelihood (ML) tree reconstructions of datasets 1 to 7 (Figure S8: dataset 1 to Figure S14: dataset 7; Figure S15: best tree of the additional partitioned analysis of dataset 5). Branches with <50% bootstrap support are shown as unresolved. Species for which new transcriptome data were generated in this study are in bold print. For details of phylogenetic tree reconstruction, see Methods section of main text. [file 1471-2148-14-52-S2.pdf]

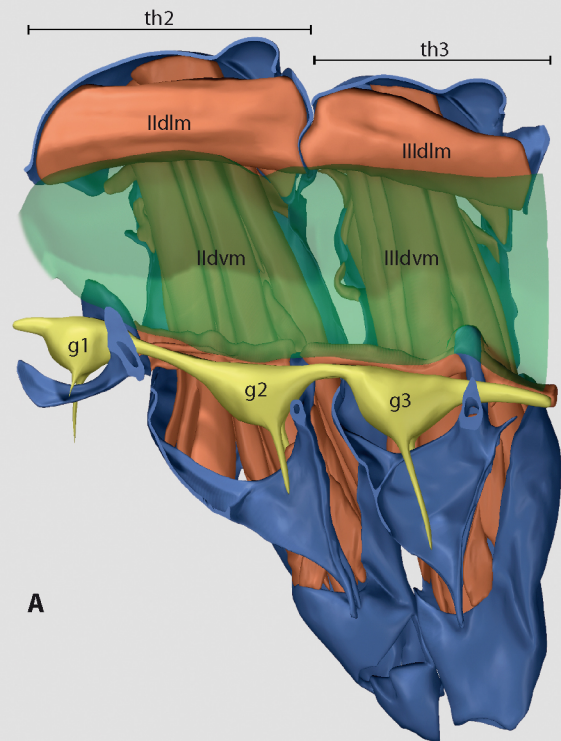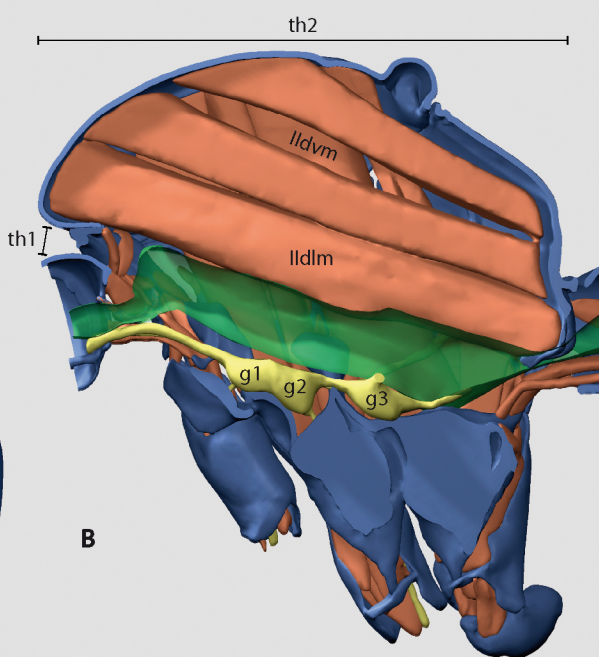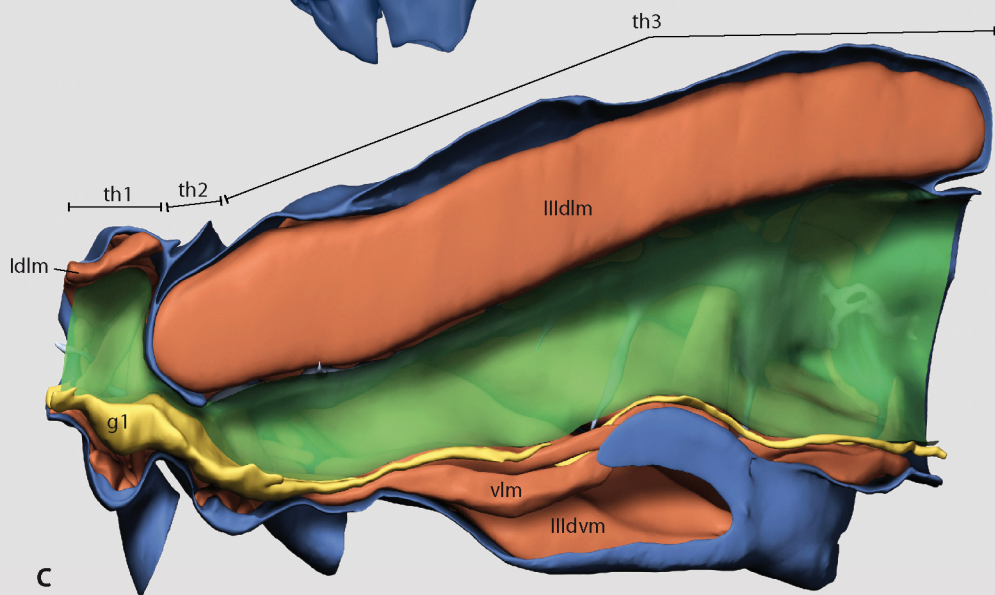

Supplement: Additional file 5: Figure_3_3D — Figure 3 of main text as 3D pdf. Click on image to activate animation. [file 1471-2148-14-52-S5.pdf]
