# Supplementary material for: The evolutionary history of holometabolous insects inferred from transcriptome-based phylogeny and comprehensive morphological data
Source: BMC Evol Biol. 2014 Mar 20;14:52. doi: 10.1186/1471-2148-14-52 (PMC4000048; doi:10.1186/1471-2148-14-52)

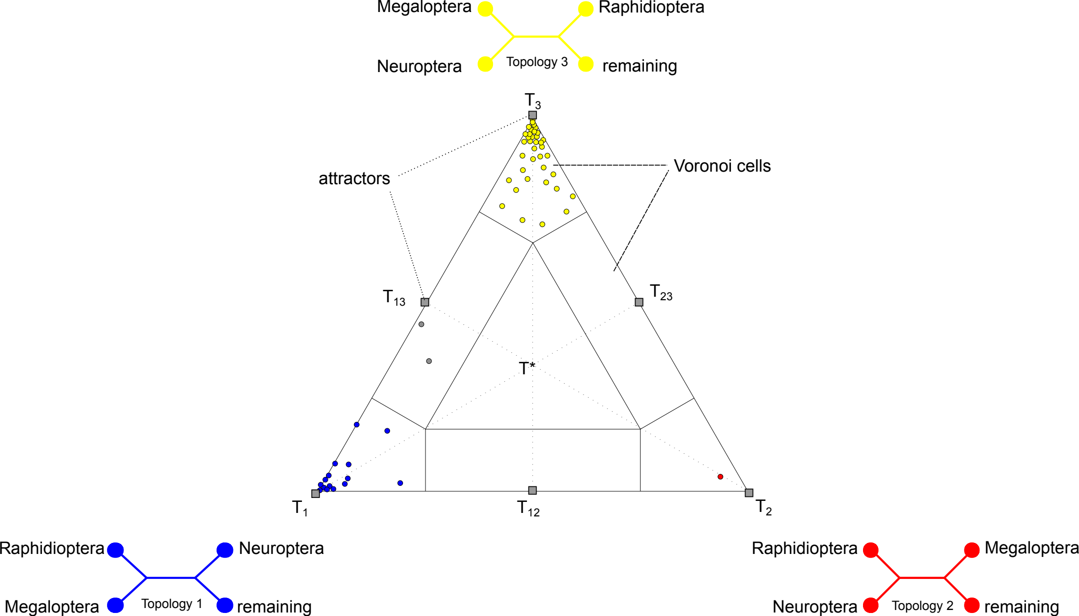
Megaloptera
Raphidioptera
Neuroptera
Topology 3
remaining
T3
attractors
Voronoi cells
T13
T23
T*
T1
T12
T2
Raphidioptera
Neuroptera
Megaloptera
Topology 1
remaining
Raphidioptera
Megaloptera
Neuroptera
Topology 2
remaining

LM_simplex

number of quartets: 142800

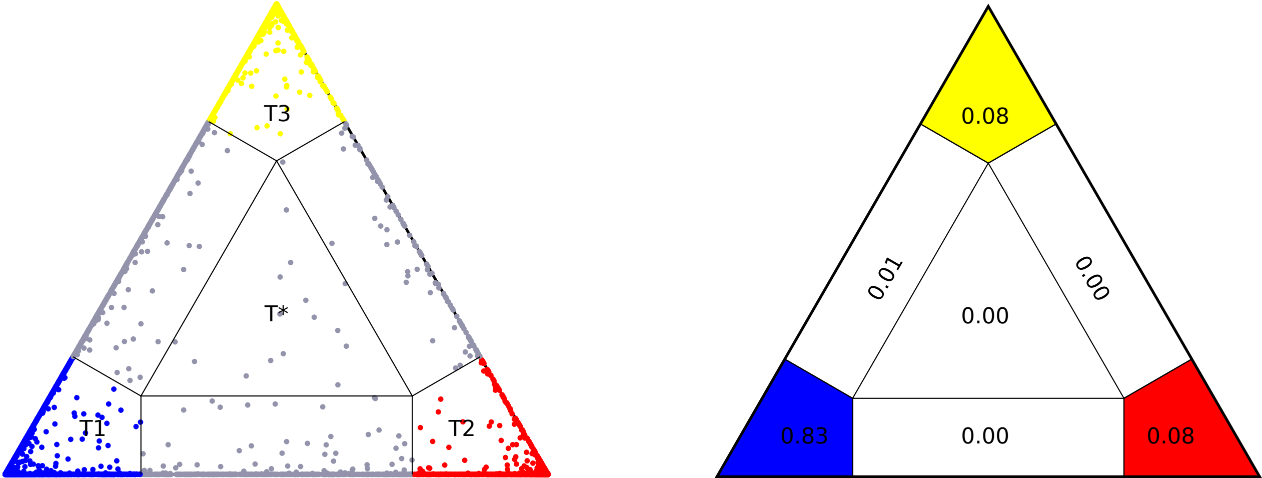

LM_simplex

number of quartets: 20160

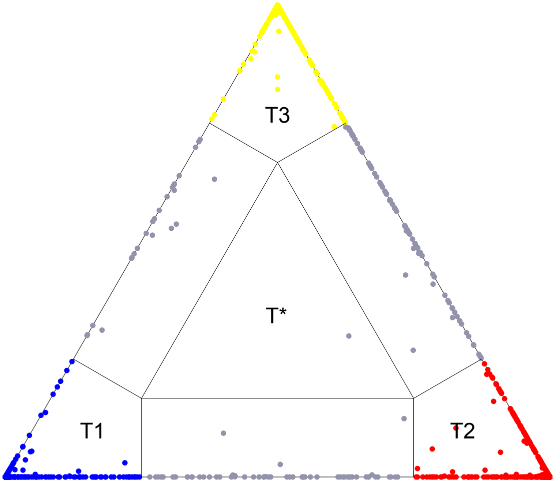

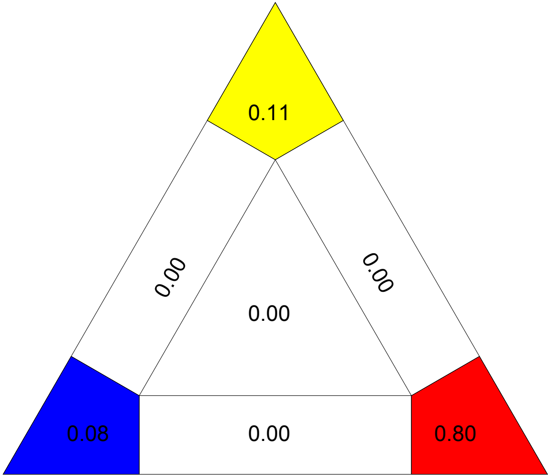

LM_simplex

number of quartets: 1

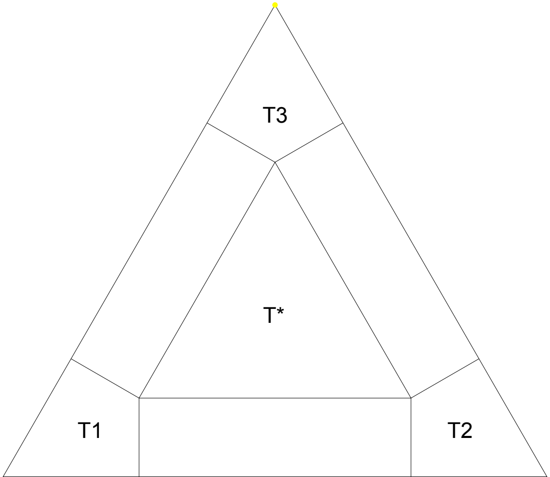

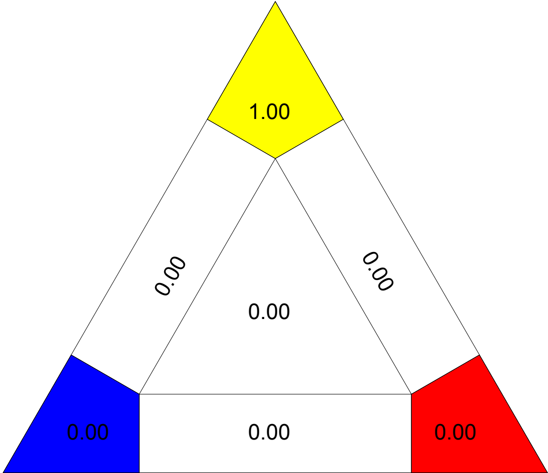

LM_simplex

number of quartets: 134

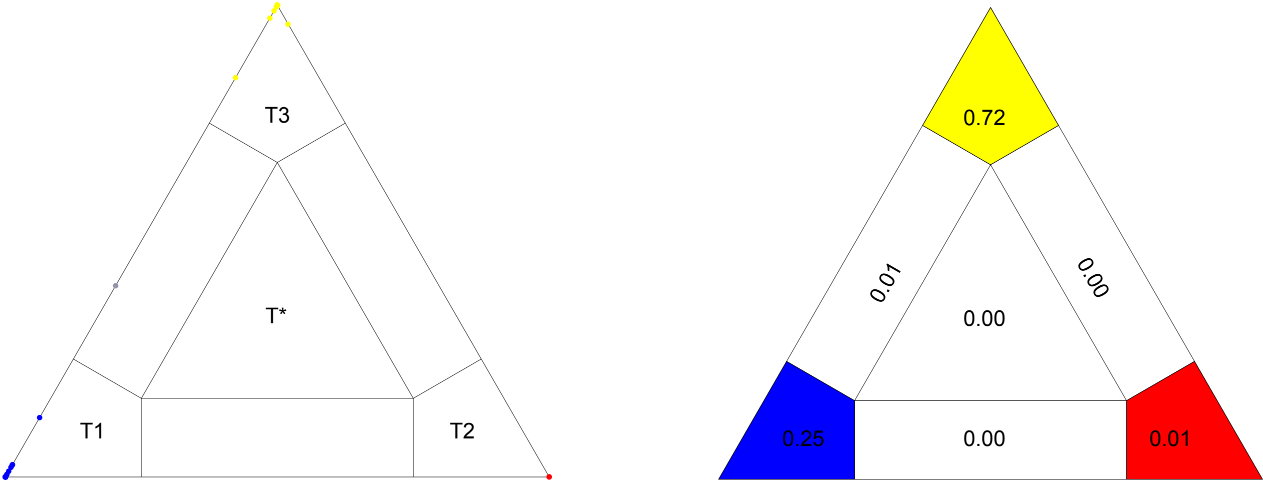

LM_simplex

number of quartets: 1220

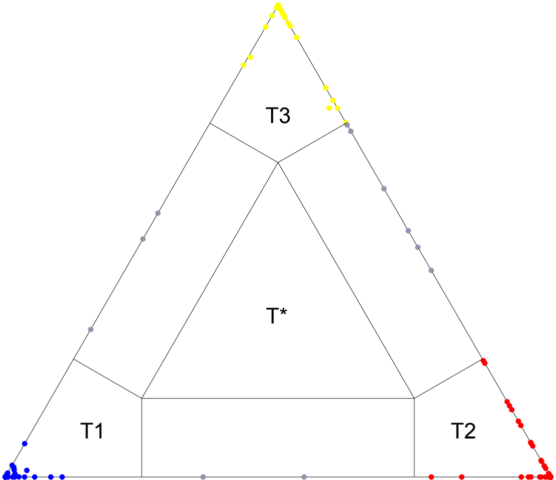

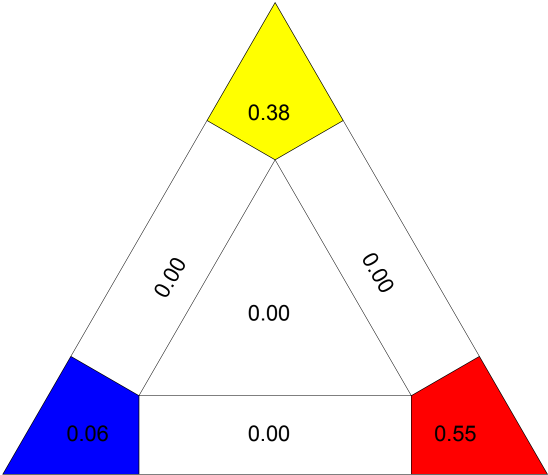

LM_simplex

number of quartets: 80640

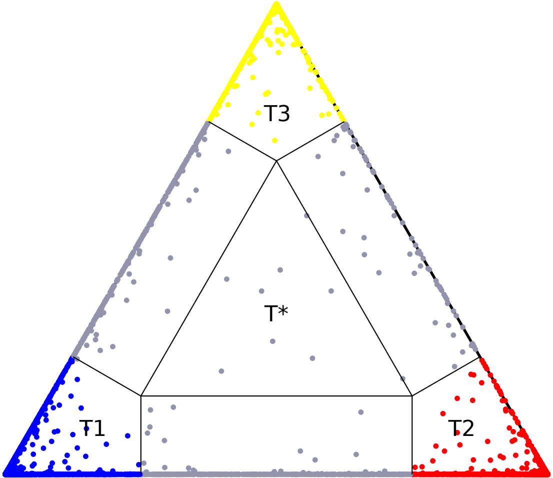

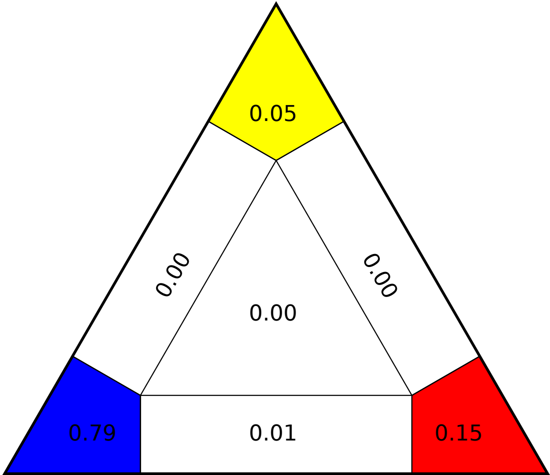

LM_simplex

number of quartets: 57600

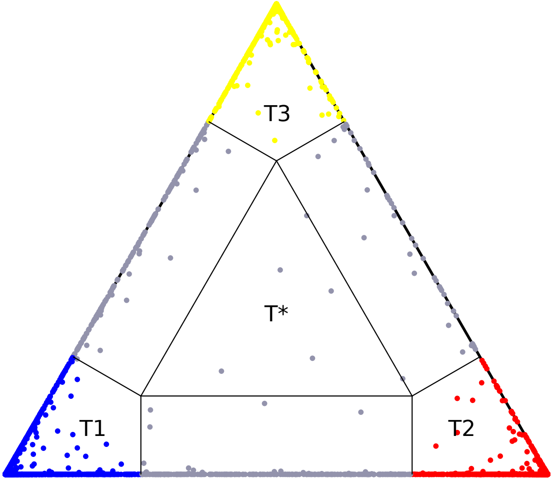

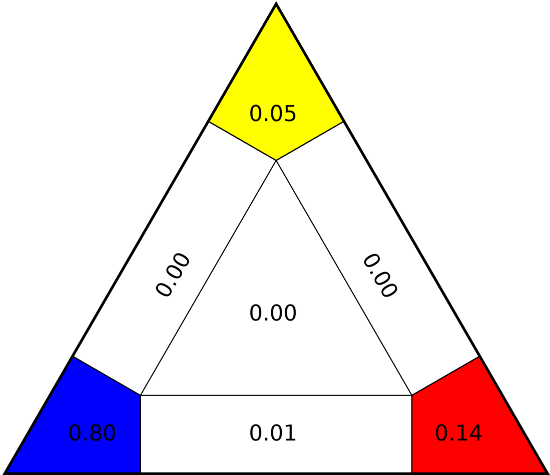

LM_simplex

number of quartets: 1034

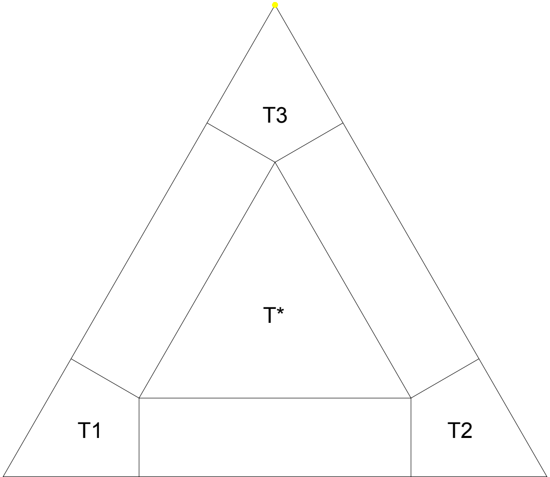

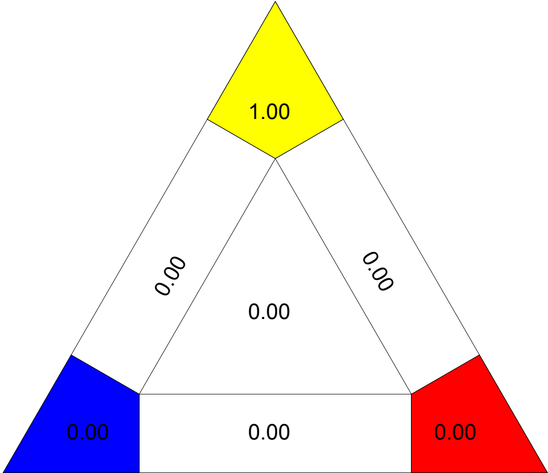

Dataset 5, 2D simplex graph, FcLM derived from partitioned analysis

LM_simplex

number of quartets: 1220

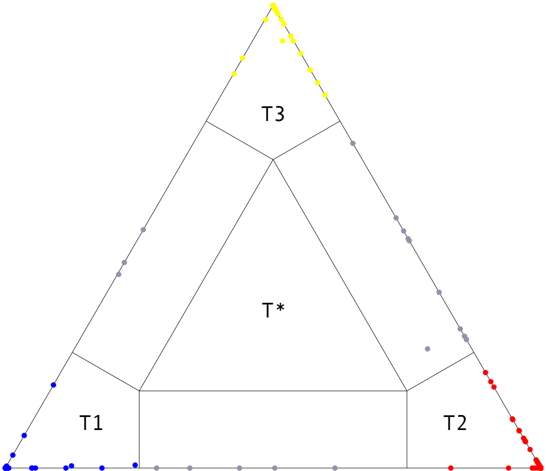

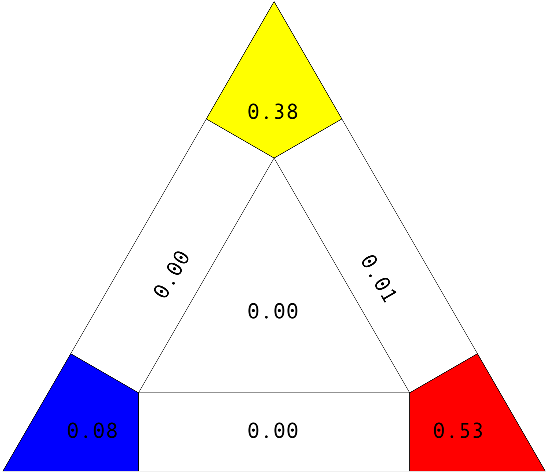

Supplement: Additional file 3: Figures S16-S25 — Results of the Four-cluster Likelihood Mapping (FcLM) as 2D simplex graphs. Figure S16. Exemplary 2D simplex graph based on the Four-cluster Likelihood Mapping (FcLM). For explanations see Additional file 4, Chapter 3. Figures S17-S25. 2D simplex graphs showing results of the Four-cluster Likelihood Mapping (FcLM) of datasets 1 to 7 (Figure S17: dataset 1 to Figure S221: dataset 5; Figure S22 and S23: dataset 6a and 6b; Figure S24: dataset 7, Figure S25: additional partitioned analysis of dataset 5). Left: the support for each quartet is shown as a single dot mapped onto the 2D simplex graph. Right: proportion of quartets with predominant support for the respective topology is given. For details on methods, topologies T1, T2, and T3, and interpretation of results see Methods and Results section of the main text, Additional file 4, Chapter 3, and Figure S16. [file 1471-2148-14-52-S3.pdf]
