# Supplementary material for: The evolutionary history of holometabolous insects inferred from transcriptome-based phylogeny and comprehensive morphological data
Source: BMC Evol Biol. 2014 Mar 20;14:52. doi: 10.1186/1471-2148-14-52 (PMC4000048; doi:10.1186/1471-2148-14-52)
Supplement: Additional file 4 — More details on methods and results. The text gives more detailed information on methods (generation of new transcriptome data and retrieval of published data, orthology assignment, and Four-cluster Likelihood Mapping), and provides additional results (rogue taxa, morphological analyses). [file 1471-2148-14-52-S4.pdf]

Additional file 4

More details on methods and results

The evolutionary history of holometabolous insects inferred from transcriptome-based phylogeny and comprehensive morphological data

Ralph S. Peters*, Karen Meusemann*, Malte Petersen, Christoph Mayer, Jeanne Wilbrandt, Tanja Ziesmann, Alexander Donath, Karl M. Kjer, Ulrike Aspöck, Horst Aspöck, Andre Aberer, Alexandros Stamatakis, Frank Friedrich, Frank Hünefeld, Oliver Niehuis, Rolf G. Beutel, and Bernhard Misof[$]

* these authors contributed equally

[$] corresponding author; Email: b.misof.zfmk@uni-bonn.de

Chapter 1: New transcriptome data and retrieval of published data

The starting material used for isolation of RNA for sequencing transcriptomes of 13 holometabolous insect species was obtained from field collections at various sites. Collection data, number, stage and sex of specimens, and further details on the material are listed in Additional file 6, Table S1.

RNA extraction, generation and normalization of cDNA libraries, sequencing and transcriptome assembly were conducted by LGC Genomics GmbH, Berlin, Germany.

RNA isolation, and normalized cDNA library construction

For all species, total RNA was isolated using a Trizol-GTI-Lithium chloride (LiCl) approach, suitable to quickly and reliably inactivate RNAses. Traces of RNAses and phenol were removed by a guanidinium-thiocyanate-isobutanol (GTI) precipitation. Contaminations of genomic DNA were removed by a subsequent LiCl precipitation. For the species Corydalinae sp. (Megaloptera), total RNA was further purified on Chromaspin 1000 Columns (clontech) to remove the majority of degraded RNA. The total RNA of the twisted winged parasite (Strepsiptera), *Mengenilla moldrzyki*, had been purified in a similar way (see Niehuis et al. 2012); for this species we used the already available cDNA library.

For the construction of normalized cDNA libraries, mRNA was purified from 2-5 µg total RNA by exonuclease digestion followed by LiCl precipitation (mRNA-Only Eucaryotic mRNA Isolation Kit, Epicentre, Madison, WI, USA). One half of the purified mRNA was used for first-strand cDNA synthesis. Synthesis and amplification of cDNA was done according to the Mint-Universal cDNA Synthesis Kit user manual (Evrogen, Moscow, Russia). 800 ng of amplified cDNA were used as starting material in the normalization reaction using the

Trimmer Kit (Evrogen, Moscow, Russia). Normalized material was re-amplified (18 PCR cycles). For size selection and cloning, 2 µg of normalized cDNA was digested with 10 Units SfiI for 2 hours at 48°C. Fragments > 800 bp were isolated from an LMP agarose gel and purified using the MinElute Gel Extraction Kit (Qiagen, Hilden, Germany). 200 ng of purified cDNA fragments were ligated to 10 ng Sfi cut and dephosphorylated pDNR-lib Vector (Clontech) in 10 µl volume using the Fast Ligation Kit (NEB, Ipswich, MA, USA). Ligations were desalted by ethanol precipitation, and re-dissolved in 10 µl water. 3 x 1.5 µl desalted ligation was used to transform NEB10b competent cells (NEB, Ipswich, MA, USA). 96 clones were chosen randomly for sequencing to verify successful normalization. Roughly a million clones were plated on LB-Cm plates, scrapped off the plates and stored as glycerol stocks at -70°C. One half of the cells were used to inoculate a 300 ml Terrific Broth/Cm culture, which was grown for 5 hours at 30°C. Plasmid DNA was prepared using standard methods (Qiagen, Hilden, Germany). 200 µg of purified plasmid DNA was digested with 100 Units SfiI for 2 hours at 48°C. cDNA inserts were gel-purified (LMP-Agarose/MinElute Gel Extraction Kit) and ligated to high-molecular-weight DNA using a proprietary Sfi-linker.

Illumina Sequencing

Library generation for the Illumina HiSeq2000 sequencing was carried out according to the Illumina TruSeq DNA sample preparation kit. In short, the concatenated inserts were sheared randomly using the Covaris AFA technology (Kbio) to fragments ranging in size from 150 bp to 250 bp. These fragments were end polished and the TruSeq adaptors containing a sample specific index were added to the ends of the fragments by ligation. Library concentration was measured using the Qubit 2.0 fluorometer and the Agilent Bioanalyzer (Invitrogen, Agilent). Four libraries each were combined in an equimolar mixture generating a library pool. Sequencing was carried out in three batches：

1) *Nevrorthus apatelios, Sialis lutaria, Micropterix calthella, Tipula maxima* (Casava 1.8)
2) *Raphidia ariadne, Priacma serrata, Xyela alpigena, Philopotamus ludificatus* (Casava 1.8)
3) *Nannochorista philpotti, Archaeopsylla erinacei, Carabus granulatus*, Corydalinae sp. (Casava 1.8.2)

For batch 1 and 2, the TruSeq PE Cluster Kit v2,5 ï cBot ï HS was used to load and hybridize each pool to 1 channel of a flow cell using the cBot (Illumina). Sequencing of 2 x 100 bp (paired-end) was performed using the TruSeq SBS Kit ï HS chemistry (200 cycles) on a HiSeq 2000 (Illumina). For batch 1, a total of 147.55 Mio. raw paired reads and for batch 2, a total of 140.23 Mio. raw paired reads with a length of 2 x 100 bp were obtained. Samples of batch 3 were analyzed in analogy using the TruSeq PE Cluster Kit v3 ï cBot ï HS and the TruSeq SBS Kit v3 ï HS (200 cycles) resulting in 218.75 Mio. raw paired reads.

Sequencing of transcriptome data of the strepsipteran species was conducted earlier (Illumina HiSeq2000, CASAVA Version 1.7, see Niehuis et al. 2012).

Assembly of sequence reads to transcripts

Prior to assembly the sequence reads underwent a demultiplexing step, a chastity and quality filtering and were screened for the Sfi-linker that was used for concatenation. The linker sequences were clipped out of the reads and the clipped reads assembled to individual transcripts using the software Newbler v. 2.5 or 2.6 (3rd batch).

Sequence of clipped out including Sfi-linker:

```
5’-CCCGGCCGTAATACGACTCACTATAGGGAGGCGGCCGTTTTTTTTTTTTTTTTTT
   GGGCCGGCATTATGCTGAGTGATATCCCTCCGCCGGCAAAAAAAAAAAAAAAAAA-5‘
```

(The 5óC stretch and the 3óT stretch can be longer)

In each batch the following cDNA library tags were used:

**batch no.**	**Genus, species**	**cDNA-library Tag**
batch 1	*Nevrorthus apatelios*	ACACAC
batch 1	*Sialis lutaria*	ACAGTC
batch 1	*Micropterix calthella*	no Tag
batch 1	*Tipula maxima*	ACATGC
batch 2	*Raphidia ariadne*	ACATGC
batch 2	*Priacma serrata*	ACACAC
batch 2	*Xyela alpigena*	ACAGTC
batch 2	*Philopotamus ludificatus*	no Tag
batch 3	*Nannochorista philpotti*	no Tag
batch 3	*Archaeopsylla erinacei*	ACACAC
batch 3	*Carabus granulatus*	ACAGTC
batch 3	Corydalinae sp.	prepared separately, no Tag necessary

The tag sequence was added to the PlugOligo3M adaptor (Mint Universal Kit, Evrogen) sequence between SfiI-Site and poly-G. Modified Plug adaptors were purchased from MWG-Biotech as HPSF purified oligos.

PlugOligo 3M adapter /no TAG:

```
5' AAGCAGTGGTATCAACGCAGAGTGGCCATTACGGCCGGGGG P 3'
```

PlugOligo 3M adapter /ACACAC:

5' AAGCAGTGGTATCAACGCAGAGTGGCCATTACGGCCACACACGGGGG P 3'

PlugOligo 3M adapter /ACAGTC:

5' AAGCAGTGGTATCAACGCAGAGTGGCCATTACGGCCACAGTCGGGGG P 3'

PlugOligo 3M adapter /ACATGC:

5' AAGCAGTGGTATCAACGCAGAGTGGCCATTACGGCCACATGCGGGGG P 3'

For statistics on the sequencing and assembly results see Additional file 7, Table S2. For more details, refer to the description deposited at the Sequence Read Archive (SRA) and Transcriptome Shotgun Assembly Database (TSA) at GenBank, NCBI.

Raw sequence reads were deposited at the NCBI Sequence Read Archive (SRA) and Transcriptome assemblies at the Transcriptome Shotgun Assembly Database (TSA), NCBI, Umbrella Bioproject ñEvolution of Holometabolaò, Bioproject ID PRJNA176423 (see Additional file 7, Table S2 and http://www.ncbi.nlm.nih.gov/bioproject).

Prior to submission to the TSA, we screened all assemblies for suspicious sequences with a local installation of VecScreen (http://www.ncbi.nlm.nih.gov/VecScreen/VecScreen.html) and the UniVec database 7.0 (http://www.ncbi.nlm.nih.gov/VecScreen/UniVec.html) for vector and linker/adapter contamination, according to the NCBI submission guidelines. Originally, these tools had been developed to exclude sequences that might originate from a vector. Since Illumina sequencing does not require cloning vectors, a positive VecScreen hit could result from foreign genetic material incorporated into the genome, such as parts of viral or bacterial genomes. Since this is not desired for orthology assignment of transcripts, we excluded such positive hits prior to submission and did not include them in the orthology assignment step (see Chapter 2). Terminal strong and moderate hits were removed. In case of internal hits the contamination was removed and the query sequence split into two separate sequences. Remaining sequences shorter than 200 bp were excluded according to the NCBI guidelines, and not considered for further analysis.

Retrieval of published data

We screened public databases (TSA, GenBank, dbEST, status: summer 2012) and downloaded all available published transcriptome assemblies of neopteran insects (hemi- and holometabolan) from various sources. For orthology assignment, we used only those assemblies with a minimum number of 3,000 contigs and only data from maximally three species per genus. Included species, source of download, the number of contigs, and other information are listed in Additional file 8, Table S3. We used available official gene sets on protein level of 14 species (13 insects, 1 crustacean species) for the compilation of an ortholog reference set (see Chapter 2). To assign respective transcripts, we additionally retrieved the OGSs on nucleotide level.

Chapter 2: Orthology assignment

Design of an ortholog reference set

We specifically selected an ortholog reference set for orthology assignment instead of using published ortholog sets (see below). This was done on the basis of the database OrthoDB4 (http://cegg.unige.ch/orthodb4/ and http://cegg.unige.ch/node/658#Arth; [60]). We compiled a set of genes that are orthologous among Pancrustacea (node selection: *Daphnia pulex* and insects) and that occur in single-copy in the genomes of each of the following 14 reference species for which a whole genome sequence was available: *Daphnia pulex*, *Acyrthosiphon pisum*, *Pediculus humanus*, *Apis mellifera*, *Nasonia vitripennis*, *Pogonomyrmex barbatus*, *Linepithema humile*, *Tribolium castaneum*, *Bombyx mori*, *Anopheles gambiae*, *Aedes aegypti*, *Culex quinquefasciatus*, *Drosophila persimilis*, and *Drosophila melanogaster* (see Additional file 9, Table S4). For these 14 taxa, we downloaded the amino acid sequences that are associated with the 1,343 identified groups of orthologous genes (OGs; a.k.a. core orthologs; [43]) from the OrthoDB database version 4 (ftp://cegg.unige.ch/OrthoDB4/). The amino acid sequences of each OG were aligned with MAFFT 6.903b [44,61] using the L-INS-i algorithm. The resulting multiple amino acid sequence alignments served as a basis to build pHMMs with the program hmmbuild from the HMMER 3.0 software package [62]. For the reverse BLAST search of candidate transcripts, we generated BLAST databases using the official gene sets of the reference species, corresponding to those in OrthoDB4, with the program makeblastdb of the NCBI BLAST 2.2.24+ software package [63]. The ortholog reference set is available upon request.

Annotations for the 1,343 ortholog groups (OGs) of the 14 reference species were obtained from OrthoDB4 in two different ways. Please note that the annotations of OGs (Table S5) are not necessarily representative and do not imply that all sequences in one group necessarily fit the annotation and thus exert the same funxtion.

For as many OGs as possible (292), annotations were obtained using a consensus rule algorithm (kindly provided by Robert Waterhouse, unpublished). For each OG, all species-specific genes for which a Swiss-Prot descriptor was available were compiled. Then, the descriptors were cleaned (*i.e.*, terms like 'putative' or 'uncharacterized' were removed) and analyzed, counting the appearances of phrases and subphrases. To assign an annotation to an OG, the following conditions had to be fulfilled: i) more than 33 % of the species-specific genes have a Swiss-Prot descriptor, ii) more than 20 % have a useful Swiss-Prot descriptor (*i.e.*, the cleaned descriptors say more than only 'protein'), and iii) the most frequently occurring phrase is contained in more than 33 % of those useful descriptors. Annotations

obtained by this method are marked with an asterisk in Table S5. OrthoDB4 only contained few genes linked to Swiss-Prot, thus this strategy did not provide annotations for all OGs.
For the remaining 1,051 OGs, annotations were assigned using a less regulated algorithm. Here, the annotation given for the gene of *Pediculus humanus* in OrthoDB4 was assigned to an OG after being cleaned (see above). Since no consensus rule was applied, cleaning could result in the complete removal of the annotation, which is indicated with an 'x' in Table S5. *P. humanus* was chosen because it was included as reference species in the used ortholog reference set, and because formulated annotations (in contrast to alphanumerical codes given for other species) are given for this species in all OGs.

Orthology assignment of transcript sequences

Orthology of transcripts was assessed with HaMStRad (Petersen, unpublished, status: March 2013) using our own ortholog reference set derived from OrthoDB 4 (see above). HaMStRad is based on the orthology assignment pipeline HaMStR ([43], see also http://www.deep-phylogeny.org/hamstr/, version 8). The modified files are available at https://github.com/mptrsen/HaMStRad. In contrast to HaMStR, HaMStRad makes use of the software *Exonerate* 2.2.0 [64] to provide frameshift-corrected nucleotide transcripts. In order to predict open reading frames, HaMStRad uses the software *Exonerate* to align nucleotide sequence transcripts that have been assigned to a given identified OG at the translational level to the amino acid sequence of the reference species that showed the highest similarity in a reciprocal BLAST search. To provide the most fitting frameshift correction, *Exonerate* was run using an alignment model specialized for protein-to-genome sequence alignments. Further changes include bug-fixes and a modified output directory structure for efficient retrieval of the relevant data. In order to obtain both the amino acid and the corresponding nucleotide sequences of those genes from the 14 reference species that are part of the 1,343 OGs, we ran HaMStRad with the predicted transcript nucleotide sequences of the reference species (see Additional file 9, Table S4) as input.
Subsequent to orthology assignment and after excluding suspicious sequences according to the UniVec database (see Chapter 1), we kept only taxa with a minimum of 200 orthologous genes for further analyses: *Coptotermes formosanus* (Isoptera), *Pachypsylla venusta* (Hemiptera), *Papilio xuthus* (Lepidoptera), *Rhynchosciara americana* and *Anopheles albimanus* (both Diptera) were excluded. All other methodological steps concerning orthology assignment are given in the main text of this paper.

Chapter 3: Four-cluster Likelihood Mapping (FcLM)

The FcLM approach [23] allows to evaluate support for a specifically addressed phylogenetic relationship, thus testing one hypothesis in contrast to a phylogenetic tree with multiple taxa, where multiple hypotheses are inherent. Applying FcLM, for each dataset, species are binned into four groups or clusters. Exemplarily, we describe the procedure for the analysis of dataset 4: With this dataset, we address how the four groups Raphidioptera, Megaloptera, Neuroptera and remaining holometabolan insects are related to each other. In the FcLM, all possible non-redundant quartets (one quartet represents a 4-taxon tree) are drawn. Each quartet must contain one representative of each of the four included taxonomic groups. For a given quartet, only three topologies are unambiguous (T_1, T_2, or T_3). In this example, T_1 supports a split between (Raphidoptera, Megaloptera) and (Neuroptera, remaining holometabolans). Thus, Raphidioptera and Megaloptera are closer related to each other than to Neuroptera or remaining holometabolans. T_2 supports a split between (Raphidioptera, Neuroptera) and (Megaloptera, remaining holometabolans), T_3 a split between (Raphidioptera, remaining holometabolans) and (Megaloptera, Neuroptera), thus Megaloptera and Neuroptera are the closest relatives. In summary, we have the following possible unambiguous topologies:

T_1: Raphidioptera, Megaloptera | Neuroptera, remaining holometabolans
T_2: Raphidioptera, Neuroptera | Megaloptera, remaining holometabolans
T_3: Raphidioptera, remaining holometabolans | Megaloptera, Neuroptera

For each dataset, we can calculate for each drawn quartet the support for each of the three topologies with a Likelihood mapping approach, *e.g.*, implemented in Tree-Puzzle or RAxML. The support values $\breve{u}_1$, $\breve{u}_2$,, $\breve{u}_3$ (Log-Likelihood values) are calculated for T_1 , T_2,, and T_3. Then, the values are transformed and relative support values (s_1, s_2, s_3) are calculated for each quartet:

$s_1 = \breve{u}_1 / (\breve{u}_1 + \breve{u}_2 + \breve{u}_3)$
$s_2 = \breve{u}_2 / (\breve{u}_1 + \breve{u}_2 + \breve{u}_3)$
$s_3 = \breve{u}_3 / (\breve{u}_1 + \breve{u}_2 + \breve{u}_3)$

The relative support values can be considered as coordinates in a 3D coordinate system. Thus they can be simply mapped into a so-called 2D simplex graph (see [23], Figure 3). The support for each quartet is mapped onto the simplex graph in one of seven possible cells (called Voronoi cells, see [23]). Quartets with predominant or maximal support for either one of the topologies T_1, T_2 or T_3 are mapped onto the Voronoi in the corners of the graph. Quartets mapped onto Voronoi cells corresponding to T_{12}, T_{13} and T_{23} (grey areas) do not have a clear support for T_1, T_2 and T_3. For quartets mapped onto T*, all topologies are equally likely. Examples: If the relative support s_1 (referring to topology T_1), s_2 (referring to topology T_2) and s_3 (referring to topology T_3) is identical, the overall support (represented by

a dot in the 2D simplex graph) is mapped exactly in the middle (T*) of the 2D simplex graph: the quartet-tree is not resolved, showing a similar support for T_1, T_2 and T_3 (see Additional file 3, Figure S15). If $s_1 = 0$, $s_2 = 0$, and $s_3 = 1$ (maximal), the dot is mapped exactly onto attractor a1: for this quartet, the support is maximal for topology T_3, *i.e.*, for Megaloptera and Neuroptera being sister groups.

For further explanations, refer to Strimmer and von Haeseler [23].

In our example of dataset 4 (Additional file 3, Figure S19), all quartets mapped onto the Voronoi cell corresponding to T_1 (blue) show predominant or maximal support for T_1. The same holds for T_2 (red) and T_3 (yellow). The majority (72 %) of all quartets drawn from dataset 4 showed predominant or maximal support for Megaloptera and Neuroptera being closest relatives (T_3), however, signal was present as well for other topologies (25 % for T_1).

The four groups (or clusters) that were selected to design our seven datasets and to perform FcLM, and the species that were assigned to each cluster are shown in Additional file 5.

Chapter 4: Rogue Taxon Analysis

We scrutinized our datasets for rogue taxa and examined to what extend they affected bootstrap support. We applied the stand-alone version of RogueNaRok [58]: taxa with unstable position in a predominantly stable topological environment are classified as rogues. In particular, we scrutinized for rogue taxa affecting either the majority-rule (MR) consensus tree, the consensus tree with the traditionally important frequency threshold of 75 %, the strict consensus tree, the greedily refined consensus tree (see [65]), or the support values on the best-known ML tree. For each combination, we varied the approximation parameter of RogueNaRok and only report the best result per dataset.

Overall, majority rule bootstrap consensus trees of the seven datasets exhibited a high degree of resolution and branch support. The proportion of support present in a bootstrap consensus tree versus the maximum support a tree with the same number of taxa could have, can be measured with the relative bipartition criterion (RBIC, [36]). For instance, the RBIC of the MR consensus trees of the dataset ranged between 90 % and 100 % and even for the very conservative strict consensus tree, RBIC values were in the range of 57.6 % and 100 %.

Since small support value improvements are of minor interest, we did not consider taxa as rogues that, if pruned from the respective dataset, improved the overall support of a tree by less than 30 %. Only two taxa in four out of the seven datasets showed considerable instability and reduce support values: Pruning *Centris flavifrons* (Hymenoptera: Apidae) from dataset 4 increased the overall support of the 75% bootstrap consensus tree and the strict bootstrap consensus tree by 1.00 (*i.e.*, by one clade with 100% bootstrap support); pruning

C. flavifrons from dataset 2 and 6 increased the support of the strict bootstrap consensus tree by 1.00. Pruning *Maconellicoccus hirsutus* (Hemiptera: Pseudococcidae) from dataset 1 increased the overall support of the strict bootstrap consensus tree by 1.00. However, these support values only refer to few internal branches (within bees, and within Sternorrhyncha) that are not in focus of our study, and refer only to the 75% bootstrap consensus or the strict bootstrap consensus tree. Concerning the phylogenetic relationships that we addressed in this study and concerning the MR bootstrap consensus trees, all our datasets were free of rogues.

Chapter 5: Morphological characters

Here, we list ancestral states and apomorphies reconstructed using Mesquite 2 [33] based on character state matrix from Beutel et al. [15]. For methods, see Methods Section in the main text. The numbers refer to the character (first number) and the character state (second number) in the data matrix from Beutel et al. [15].

Apomorphic character states are marked with * (as, *e.g.*, 138.1 see below).

Plesiomorphic characters states have no symbol (as, *e.g.*, *1.0.*, see below).

Character states for which it can not be decided whether they are plesiomorphic or apomorphic (polarity) are marked with ? (as, *e.g.*, 26.0, see below).

A question mark in parentheses (?) after the description highlights character states that are most parsimonious for the respective clade but imply seemingly unlikely reversals in subgroups, and are therefore considered doubtful (as, *e.g.*, 100.1., see below).

For characters listed separately as ñambiguous charactersò, the groundplan character state is unclear.

Holometabola (groundplan)

1.0. Larval head orthognathous
5.1. Larval compound eyes simplified but present
6.0. Less than 15 retinula cells in ommatidia of larvae
7.0. Paired ocelli absent in larvae
(also missing in acercarian nymphs, possible apomorphy of Eumetabola)
11.0. Larval occipital furrow absent
15.1. Larval dorsal tentorial arm well developed
16.1. Larval tentorium X-shaped
20.1. Larval M. frontolabralis present
26.0. ? Intrinsic larval antennal muscles (Mm. scapopedicellaris) absent
41.0. ? Larval M. praementoparaglossalis absent

42.0. ? Larval M. praementoglossalis absent

50.0. Retractile larval abdominal prolegs absent

53.1. * Larval cerci absent (possible reversal in Strepsiptera [homology uncertain])

62.0. Adult head orthognathous

98.0. Paraglossae present

99.0. M. praementoparaglossalis present

100.0. M. praementoglossalis present

129.0. Membranous area between mesoscutellum and mesopostnotum present

131.0. Mesothoracic postalar bridge present

138.1. * Mesosternum invaginated

142.1. Mesospina present

144.1. * Mesocoxae closely adjacent medially

148.1. Mesotrochantin small

158.1. * Metasternum invaginated

162.1. * Ventral metasternal process (below metafurca) forming sterno-coxal joint present

165.1. Metatrochantin present and exposed

167.1. * Metacoxae closely adjacent medially

168.0. Metacoxal meron absent

174.1. Less than 5 costal crossveins (reversal in Neuropterida)

178.1. M. profurca-phragmalis present

180.1. M. pronoto-coxalis posterior present

190.1. M. profurca-mesofurcalis present (absent in Diptera)

191.1. M. prospina-mesofurcalis present (independently lost several times)

193.1. M. prospina-coxalis present (independently lost several times)

195.1. M. mesonoto-sternalis present (independently lost several times)

196.1. M. mesonoto-coxalis posterior present (independently lost several times)

197.1. M. mesonoto-trochanteralis present (independently lost several times)

198.1. M. mesofurca-phragmalis present

200.1. M. prophragma-mesanepisternalis moderately sized

201.0. M. prophragma-mesanepisternalis with only 1 bundle

202.1. M. mesonoto-basalaris present

207.1. M. mesonoto-pleuralis medialis large

208.0. M. mesonoto-pleuralis posterior absent

211.0. Insertion of M. mesonoto-pleuralis on posterolateral mesoscutal rim

212.1. M. mesanepisterno-axillaris present (absent in Strepsiptera)

213.1. M. mesepimero-subalaris present

215.1. M. mesopleura-sternalis present (reduced in several groups)

216.1.	M. mesofurca-pleuralis present (absent in Strepsiptera and some others)
219.1.	Insertion of M. mesobasalare-trochantinalis on the anterior mesocoxal rim
220.1.	M. mesanepisterno-coxalis posterior present (reduced in several groups)
221.1.	M. mesospina-metafurcalis present (reduced in several groups)
222.1.	* M. metascutello-postnotalis present (absent in Coleopterida and Antliophora, potential apomorphies of these lineages but ambiguous)
223.1.	M. metanoto-sternalis present (absent in Lepidoptera)
224.1.	M. metanoto-coxalis posterior present
225.1.	M. metafurca-phragmalis present
226.1.	M. metanoto-basalaris present (reduced in several groups)
232.1.	M. metanepisterno-axillaris present (absent in Diptera)
239.0.	? Arolium absent (polarity interpretation depends strongly on outgroup, arolium present in four lineages)
353.1.	* Appearance of fully developed compound eyes including external apparatus (reversal in Strepsiptera)
354.1.	* No external wing buds in immature stages (partial reversal in Strepsiptera)

Ambiguous holometabolan characters

80.	Mandibular mola present, reduced or absent?
81.	Maxillolabial complex present or absent?
102.	Sclerotized sitophore plate present or absent?
120.	Profurcal arm and propleura specifically articulated or fused?
126.	Relative size of pterothoracic segments?
350.	Number of Malpighian tubules (more than 20 or 8 or less)?

Apomorphic characters of Hymenoptera (selection)

11.1.	* Larval occipital furrow present (parallel evolution in Mecoptera)
32.1.	* Larval maxillolabial complex present (parallel evolution in Mecoptera and Amphiesmenoptera)
43.3.	* Apical prelabial region together with hypopharynx forming a compact lobe with salivary duct opening (parallel evolution in Amphiesmenoptera)
50.1.	* Retractile larval abdominal prolegs (segments II-VII) present
65.1.	* Head concave posteriorly
68.1.	* Clypeus inflected
69.1.	* Posterior tentorium massive, collar-like
96.1.	* Secondary subdivision of apical labial palpomere
103.1.	* Epipharyngopharyngeal lobe reaching into pharynx posteriorly

124.2. * Antenna-cleaning apparatus of foreleg formed by an apical tibial spur and the inner side of the pro-basitarsus

147.0. * Mesotrochantin absent

176.1. * Hamuli

237.1. * Muscle between second abdominal sternum and metacoxa present

299.1. * Close association between metapostnotum and tergum I

356.1. * Haplo-diploidy

Apomorphic characters of Aparaglossata (*i.e.*, Holometabola excluding Hymenoptera)

1.1. * Larval head prognathous

5.3. * Well-developed larval stemmata

16.0. * Larval tentorium H-shaped

98.1. * Paraglossae vestigial or absent, without muscles

99.0. * M. praementoparaglossalis absent

100.1. * M. praementoglossalis absent (reversal in basal Lepidoptera) (?)

207.0. * M. mesonoto-pleuralis medialis moderately sized

302.1. * Ventral sclerites of segment VIII (gonocoxae and gonapophyses) indistinct (reversals within Neuropterida)

Ambiguous aparaglossatan characters

142. Mesospina present or absent?

146. Mesocoxal meron present or absent?

168. Metacoxal meron absent or present?

Apomorphic characters of Neuropteroidea

62.1. * Adult head prognathous or slightly inclined (groundplan, several reversals)

Apomorphic characters of Neuropterida

6.1. * More than 15 retinula cells in stemmata of larvae

148.1. * Mesotrochantin well developed (polarity?)

153.1 * Metapostnotum medially divided

174.1. * More than 5 costal crossveins

219.0. * Insertion of M. mesobasalare-trochantinalis on the trochantin (also in Panorpidae)

227.1. * Insertion of M. metanoto-basalaris on ventrolateral part of basalar disc

235.1. * Insertion of M. metabasalare-trochantinalis on trochantin (also in Panorpidae)

238.1. * Furcostigmal muscle between metafurcal arm and first abdominal stigma present

340.1. * Trichobothria field on tergum X present

Apomorphic characters of Neuroptera + Megaloptera

134.1. * Mesothoracic prealare present (also in Amphiesmenoptera)
164.1. * Muscular connection between metafurcal arm and epimeral apophysis
348.2. ? Accumulated trichobothria on anal segment forming a rosette (groundplan of Neuropterida ambiguous)

Apomorphic characters of Megaloptera

23.1. * Sensorium on antepenultimate larval antennomere
44.2. * Larval salivary duct strongly narrowed, without recognisable lumen
52.1. * Setiferous lateral abdominal gills present in larvae

Apomorphic characters of Coleopterida

20.0. * Larval M. frontolabralis absent (parallel evolution in Neuroptera)
76.1. * Antenna with 9 flagellomeres or less
116.1. * Pronotum and propleuron partly or completely connected (also in Diptera)
120.0. * Profurcal arm and propleura not connected
127.2. * Hind wings distinctly larger than fore wings
128.1. * Median mesonotal suture absent (parallel evolution in Antliophora)
129.1. * Membranous area between mesoscutellum and mesopostnotum present
140.0. * Ventral mesosternal process (below mesofurca) forming sterno-coxal joint missing
182.0. * Ventral metasternal process (below metafurca) forming sterno-coxal joint absent (secondarily reduced)
202.1. * M. mesonoto-basalaris absent

Apomorphic characters of Mecopterida

15.1. * Larval dorsal tentorial arm strongly reduced or absent
22.5. * Less than 3 larval antennomeres (reversal to 3 in some groups)
35.1. * Larval galea and lacinia extensively or completely fused (also missing as separate structures in Neuroptera and Strepsiptera)
37.1. * Larval M. craniocardinalis absent (parallel loss in several groups)
39.1. * Larval M. craniodististipitalis present
40.0. * Larval M. submentopraementalis absent (parallel loss in several groups)
163.1. * Ventral metasternal process elongated
196.0. * M. mesonoto-coxalis posterior absent (reversal in Agathiphagidae) (?)
224.0. * M. metanoto-coxalis posterior absent (reversal in Agathiphagidae) (?)

Ambiguous mecopterid characters

141. Ventral mesosternal process short or elongated?

211. Insertion of M. mesonoto-pleuralis on posterior mesonotal process or 4th axillary sclerite?

Apomorphic characters of Amphiesmenoptera

32.1.	* Larval maxillolabial complex present
43.3.	* Apical prelabial region together with hypopharynx forming a compact lobe with salivary duct opening (parallel evolution in Hymenoptera)
125.1.	* Movable epiphysis of fore tibia present
134.1.	* Mesothoracic prealare present (also in Neuroptera + Megaloptera)
139.1.	* Mesofurca and mesepimeron fused
159.1.	* Metafurca and metepimeron fused
217.1.	* Origin of M. mesofurca-pleuralis with a tendon
258.0.	* Distance of the articulation of the distal area of axillary 3 from the notal margin at least 2x the maximum width of axillary 1
298.1.	* Field of sensilla trichodea in membrane between subalare and dorsal epimeral margin present
309.0.	* Ventral sclerites of segment IX absent
320.1.	* Extrusion of terminal segments by muscle force
325.1.	* Spermathecal gland present
326.1.	* Bursa copulatrix present
355.1.	* Male sex chromosomes homogametic

Apomorphic characters of Antliophora

90.1.	* M. craniocardinalis absent in adults
95.2.	* Two or less labial palpomeres (reversal in Siphonaptera)
104.1.	* M. clypeopalatalis enlarged (?)
105.1.	* Transverse muscle of the epipharynx absent
109.1.	* Brain and suboesophageal complex forming compact mass around pharynx
122.0.	* Prospina absent (reversal in Nannochorista) (?)
128.1.	* Median mesonotal suture absent (parallel evolution in Coleopterida)
145.1.	* Mesothoracic sternocoxale continuous with posterior eucoxa, articulated with anterior part (also in Tipulidae)
198.0.	* M. mesofurca-phragmalis present (also missing in Micropterigidae)
200.1.	* M. prophragma-mesanepisternalis enlarged

201.1. * Two bundles of M. prophragma-mesanepisternalis

208.0. * M. mesonoto-pleuralis posterior present (also in some other groups)

225.1. * M. metafurca-phragmalis absent membrane (absent in Culicidae)

Ambiguous antliophoran characters

107. Postcerebral pharyngeal pumping apparatus present or absent?

136. Elongated ventral process of mesothoracic pleural ridge present or absent (probably present, missing only in Siphonaptera)?

Apomorphic characters of Mecoptera + Siphonaptera

188.2. * M. profurca-spinalis present, connecting profurcal arms

349.1. * Acanthae of proventriculus close-set, prominently elongated

Additional References

60. Waterhouse RM, Zdobnov EM, Tegenfeldt F, Li J, Kriventseva EV: **OrthoDB: the hierarchical catalog of eukaryotic orthologs in 2011.** *Nucleic Acids Res* 2011, **39:**D283ï 288.

61. Katoh K, Misawa K, Kuma K, Miyata T: **MAFFT: a novel method for rapid multiple sequence alignment based on fast Fourier transform.** *Nucleic Acids Res* 2002, **30:**3059ï 3066.

62. Eddy SR: **Accelerated profile HMM searches.** *PLoS Comp Biol* 2011, **7:**e1002195.

63. Camacho C, Coulouris G, Avagyan V, Ma N, Papadopoulos J, Bealer K, Madden TL: **BLAST+: architecture and applications.** *BMC Bioinforma* 2009, **10:**421.

64. Slater GSC, Birney E: **Automated generation of heuristics for biological sequence comparison.** *BMC Bioinforma* 2005, **6:**31.

65. Bryant D: **A classification of consensus methods for phylogenetics.** *DIMACS series in discrete mathematics and theoretical computer science* 2003, **61:**163ï 184.
