# Supplementary material for: The evolutionary history of holometabolous insects inferred from transcriptome-based phylogeny and comprehensive morphological data
Source: BMC Evol Biol. 2014 Mar 20;14:52. doi: 10.1186/1471-2148-14-52 (PMC4000048; doi:10.1186/1471-2148-14-52)

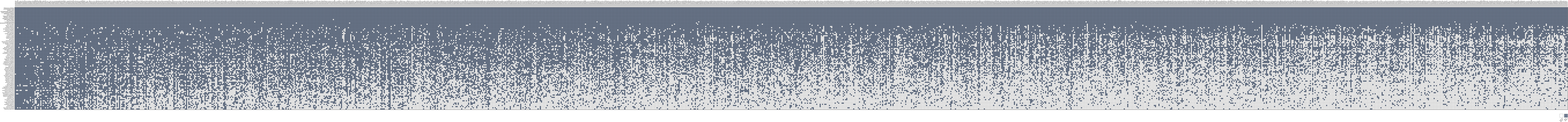

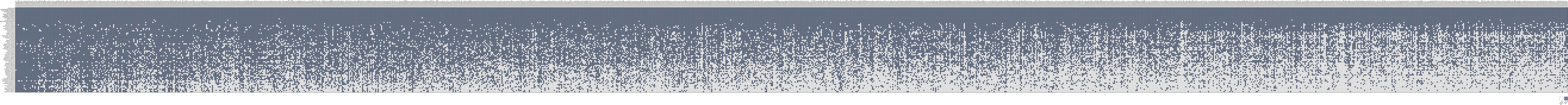
BOMBYX_MORI
POGONOMYRMEX_BARBATUS
CULEX_QUINQUEFASCIATUS
ANOPHELES_GAMBIAE
AEDES_AEGYPTI
TRIBOLIUM_CASTANEUM
NASONIA_VITRIPENNIS
DROSOPHILA_PERSIMILIS
LINEPITHEMA_HUMILE
APIS_MELLIFERA
Drosophila_sechellia
DROSOPHILA_MELANOGASTER
Bombus_terrestris
Aedes_albopictus
Anopheles_funestus
Nannochorista_philpotti
Bombus_impatiens
Archaeopsylla_erinacei
Carabus_granulatus
Corydalinae_sp
Eulaema_nigrita
Euglossa_cordata
Centris_flavifrons
Dendroctonus_ponderosae
Sarcophaga_crassipalpis
Exoneura_robusta
Apis_florea
Raphidia_ariadne
Glossina_morsitans
Manduca_sexta
Mengenilla_moldrzyki
Melipona_quadrifasciata
Micropterix_calthella
Rhagoletis_pomonella
Philopotamus_ludificatus
Priacma_serrata
Tipula_maxima
Danaus_plexippus
Ceratitis_capitata
Megachile_rotundata
Heliothis_virescens
Lutzomyia_longipalpis
Phlebotomus_papatasi
Copidosoma_floridanum
Frieseomelitta_varia
Sialis_lutaria
Megalopta_genalis
Striacosta_albicosta
Nevrorthus_apatelios
Bicyclus_anynana
Solenopsis_invicta
Armigeres_subalbatus
Teleopsis_dalmanni
Haematobia_irritans
Diabrotica_virgifera
Antheraea_assama
Bombus_insularis
Xyela_alpigena
Nasonia_giraulti
Spodoptera_frugiperda
Choristoneura_fumiferana
Cochliomyia_hominivorax
Samia_cynthia_ricini
Lysiphlebus_testaceipes
Trichoplusia_ni
Chironomus_tentans
Heliconius_erato
Plodia_interpunctella
Anopheles_stephensi
Ostrinia_nubilalis
Mayetiola_destructor

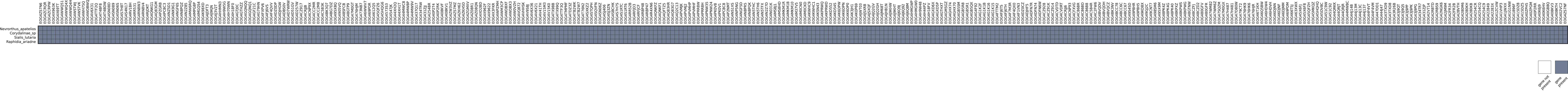
Nevrorthus_apatelios
Corydalinae_sp
Sialis_lutaria
Raphidia_ariadne
gene not present
gene present

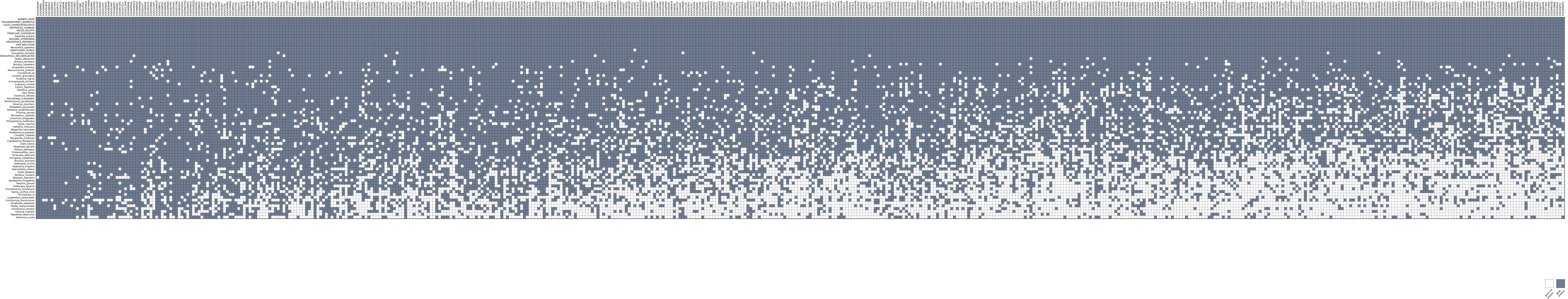
BOMBYX_MORI
POGONOMYRMEX_BARBATUS
CULEX_QUINQUEFASCIATUS
ANOPHELES_GAMBIAE
AEDES_AEGYPTI
TRIBOLIUM_CASTANEUM
Raphidia_ariadne
NASONIA_VITRIPENNIS
DROSOPHILA_PERSIMILIS
APIS_MELLIFERA
Nevrorthus_apatelios
LINEPITHEMA_HUMILE
Drosophila_sechellia
DROSOPHILA_MELANOGASTER
Aedes_albopictus
Bombus_terrestris
Bombus_impatiens
Anopheles_funestus
Nannochorista_philpotti
Corydalinae_sp
Carabus_granulatus
Eulaema_nigrita
Archaeopsylla_erinacei
Euglossa_cordata
Centris_flavifrons
Manduca_sexta
Apis_florea
Exoneura_robusta
Sarcophaga_crassipalpis
Dendroctonus_ponderosae
Glossina_morsitans
Rhagoletis_pomonella
Melipona_quadrifasciata
Priacma_serrata
Micropterix_calthella
Lutzomyia_longipalpis
Philopotamus_ludificatus
Tipula_maxima
Heliothis_virescens
Megachile_rotundata
Phlebotomus_papatasi
Ceratitis_capitata
Mengenilla_moldrzyki
Copidosoma_floridanum
Sialis_lutaria
Megalopta_genalis
Danaus_plexippus
Frieseomelitta_varia
Striacosta_albicosta
Armigeres_subalbatus
Bicyclus_anynana
Solenopsis_invicta
Diabrotica_virgifera
Haematobia_irritans
Xyela_alpigena
Bombus_insularis
Teleopsis_dalmanni
Spodoptera_frugiperda
Nasonia_giraulti
Antheraea_assama
Choristoneura_fumiferana
Samia_cynthia_ricini
Trichoplusia_ni
Lysiphlebus_testaceipes
Cochliomyia_hominivorax
Anopheles_stephensi
Plodia_interpunctella
Chironomus_tentans
Ostrinia_nubilalis
Mayetiola_destructor
Heliconius_erato
EOG4Q83HR
EOG4J9KJN
EOG4WWQ4X
EOG4PC8C8
EOG4NK9G1
EOG498SM1
EOG4V15M3
EOG40P2T4
EOG4254CQ
EOG43J9QW
EOG4SN089
gene not present
gene present

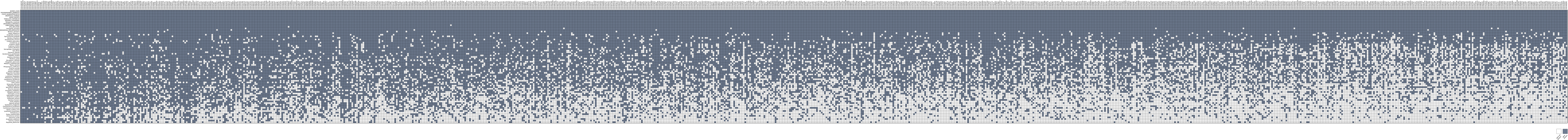

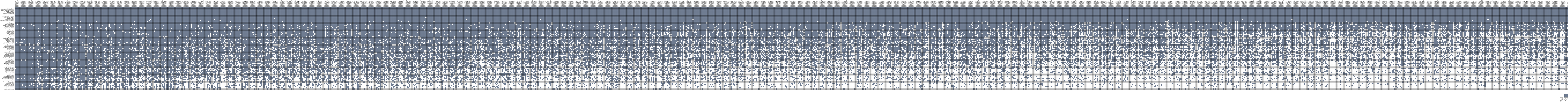
BOMBYX_MORI
POGONOMYRMEX_BARBATUS
CULEX_QUINQUEFASCIATUS
ANOPHELES_GAMBIAE
AEDES_AEGYPTI
TRIBOLIUM_CASTANEUM
NASONIA_VITRIPENNIS
DROSOPHILA_PERSIMILIS
LINEPITHEMA_HUMILE
APIS_MELLIFERA
Drosophila_sechellia
DROSOPHILA_MELANOGASTER
Bombus_terrestris
Aedes_albopictus
Anopheles_funestus
Nannochorista_philpotti
Bombus_impatiens
Archaeopsylla_erinacei
Carabus_granulatus
Eulaema_nigrita
Corydalinae_sp
Euglossa_cordata
Centris_flavifrons
Apis_florea
Exoneura_robusta
Dendroctonus_ponderosae
Sarcophaga_crassipalpis
Glossina_morsitans
Raphidia_ariadne
Manduca_sexta
Mengenilla_moldrzyki
Melipona_quadrifasciata
Micropterix_calthella
Rhagoletis_pomonella
Philopotamus_ludificatus
Tipula_maxima
Priacma_serrata
Danaus_plexippus
Megachile_rotundata
Ceratitis_capitata
Heliothis_virescens
Lutzomyia_longipalpis
Phlebotomus_papatasi
Copidosoma_floridanum
Frieseomelitta_varia
Sialis_lutaria
Megalopta_genalis
Striacosta_albicosta
Nevrorthus_apatelios
Bicyclus_anynana
Solenopsis_invicta
Armigeres_subalbatus
Teleopsis_dalmanni
Haematobia_irritans
Diabrotica_virgifera
Antheraea_assama
Bombus_insularis
Xyela_alpigena
Nasonia_giraulti
Spodoptera_frugiperda
Choristoneura_fumiferana
Cochliomyia_hominivorax
Samia_cynthia_ricini
Lysiphlebus_testaceipes
Trichoplusia_ni
Heliconius_erato
Chironomus_tentans
Plodia_interpunctella
Anopheles_stephensi
Ostrinia_nubilalis
Mayetiola_destructor
gene not present
gene present

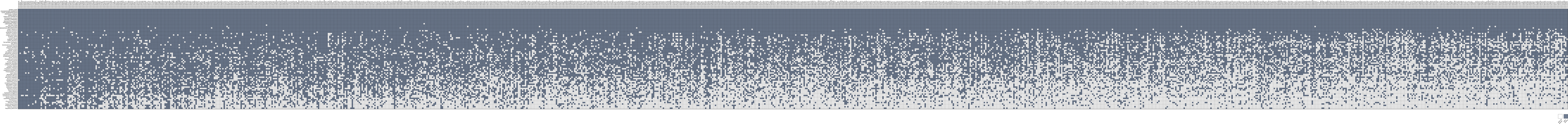

Supplement: Additional file 1: Figures S1-S7 — Presence and absence of genes in datasets 1 to 7. Files visualize the data matrices of datasets 1 to 7, in terms of gene coverage (Figure S1: dataset 1 to Figure S7: dataset 7). Grey dot: gene present. White dot: gene absent. The data matrices were visualized with mare[49]. [file 1471-2148-14-52-S1.pdf]
